# Fibroblast rejuvenation by mechanical reprogramming and redifferentiation

**DOI:** 10.1073/pnas.1911497117

**Published:** 2020-04-29

**Authors:** Bibhas Roy, Luezhen Yuan, Yaelim Lee, Aradhana Bharti, Aninda Mitra, G. V. Shivashankar

**Affiliations:** ^a^Mechanobiology Institute, National University of Singapore, 117411 Singapore;; ^b^Institute of Molecular Oncology, Italian Foundation for Cancer Research, 20139 Milan, Italy;; ^c^Division of Biology and Chemistry, Paul Scherrer Institut, 5232 Villigen, Switzerland;; ^d^Department of Health Sciences and Technology, ETH Zurich, 8092 Zurich, Switzerland

**Keywords:** lateral confinement, stem-cell-like state, redifferentiation, rejuvenation, engineered 3D tissue

## Abstract

Over the course of the aging process, fibroblasts lose contractility, leading to reduced connective-tissue stiffness. A promising therapeutic avenue for functional rejuvenation of connective tissue is reprogrammed fibroblast replacement, although major hurdles still remain. Toward this, we recently demonstrated that the laterally confined growth of fibroblasts on micropatterned substrates induces stem-cell-like spheroids. In this study, we embedded these partially reprogrammed spheroids in collagen-I matrices of varying densities, mimicking different three-dimensional (3D) tissue constraints. In response to such matrix constraints, these spheroids regained their fibroblastic properties and sprouted to form 3D connective-tissue networks. Interestingly, we found that these differentiated fibroblasts exhibit reduced DNA damage, enhanced cytoskeletal gene expression, and actomyosin contractility. In addition, the rejuvenated fibroblasts show increased matrix protein (fibronectin and laminin) deposition and collagen remodeling compared to the parental fibroblast tissue network. Furthermore, we show that the partially reprogrammed cells have comparatively open chromatin compaction states and may be more poised to redifferentiate into contractile fibroblasts in 3D-collagen matrix. Collectively, our results highlight efficient fibroblast rejuvenation through laterally confined reprogramming, which has important implications in regenerative medicine.

## Significance

The mechanical reprogramming of fibroblasts, followed by their redifferentiation into rejuvenated fibroblasts in an optimized 3D collagen matrix, made these cells more contractile and more efficient at synthesizing matrix components including laminin, fibronectin, and collagen-IV. Moreover, the rejuvenated fibroblasts obtained through this approach exhibited a decrease in DNA damage. The rejuvenated fibroblasts derived from this method precisely align into tissue architectures, suggesting its potential application as clinical implants in tissue engineering and regenerative medicine.

Fibroblasts are vital constituents of the connective tissue, which provide mechanical strength and maintain tissue homeostasis by promoting extracellular matrix (ECM) remodeling (1, 2). During aging, fibroblasts reduce their actomyosin contractility and matrix remodeling efficiencies (3–6). Transplanting of stem cells and induced pluripotent stem cells (iPSCs) are being seen as potential cellular-therapy models for rejuvenating fibroblast function (7–9). However, these interventions not only rejuvenate, but have been found to acquire genomic mutations that may increase the oncogenic potential of the proliferative fibroblasts, and major efforts are underway to improve the limitations of such methods (10). Therefore, for therapeutic purposes, it would also be ideal to rejuvenate fibroblasts using nongenetic methods.

Recently, we showed that sustained, laterally confined growth of fibroblasts on micropatterned substrates induced their reprogramming into stem-cell-like cells, even in the absence of any genetic or biochemical interventions (11). Such partially reprogrammed cells (PRs) not only exhibited stem-cell-like characteristics, but also retained their differentiation states to some extent, making them a potential model for fibroblast rejuvenation in connective tissues. As a major constituent of connective tissue, collagen-I concentration primarily regulates matrix stiffness and controls cellular processes such as contraction, adhesion, and migration via its interaction with fibroblasts (12). Furthermore, in a three-dimensional (3D) gel, matrix fibers are intertwined into a mesh-like structure, and the porosity of the mesh regulates initial cell spreading and migration through the entangled fibrils (13). Therefore, 3D collagen matrices with appropriate steric (porosity) and mechanical (stiffness) features that closely resemble fibrous connective tissue will be ideal for exploring the fate of reprogrammed cells in a tissue-like microenvironment (14, 15).

In this paper, we describe a unique method of fibroblast rejuvenation, which involves partial reprogramming of fibroblasts by growing them under lateral confinement, followed by their redifferentiation into fibroblast-like cells by embedding them in a 3D collagen-I matrix. We optimized an appropriate 3D collagen matrix density for the redifferentiation process. Here, we demonstrate fibroblast rejuvenation by revealing enhanced actomyosin contractility, collagen remodeling, and matrix protein deposition in redifferentiated cells compared to parent fibroblasts. RNA sequencing (RNA-seq) reveals a shift in transcriptome from a fibroblastic to an intermediate reprogrammed state following lateral confinement, which shifts back to the fibroblastic transcriptome (enhanced expression of genes related to contractile cytoskeletal pathways) upon redifferentiation in the collagen matrix. Importantly, we show an amelioration of DNA damage, which was facilitated by an increase in laminA levels in the nucleus upon rejuvenation. In terms of changes to nuclear architecture, we reveal that the comparatively open chromatin compaction state (chromatin poise state) induced by partial reprogramming was more likely to differentiate into contractile fibroblasts in response to ECM cues present in the 3D collagen matrix than the parental fibroblasts. In summary, we suggest that the mechanically induced partial reprogramming approach described here can overcome the shortcomings of conventional rejuvenation methods, including generation of short-lived or oncogenic fibroblasts, and therefore could have potential implications in the field of regenerative medicine.

## Results

### Redifferentiation of Fibroblasts from Partially Reprogrammed Spheroids Depends on 3D Collagen Matrix Density.

**Fig. 1.**
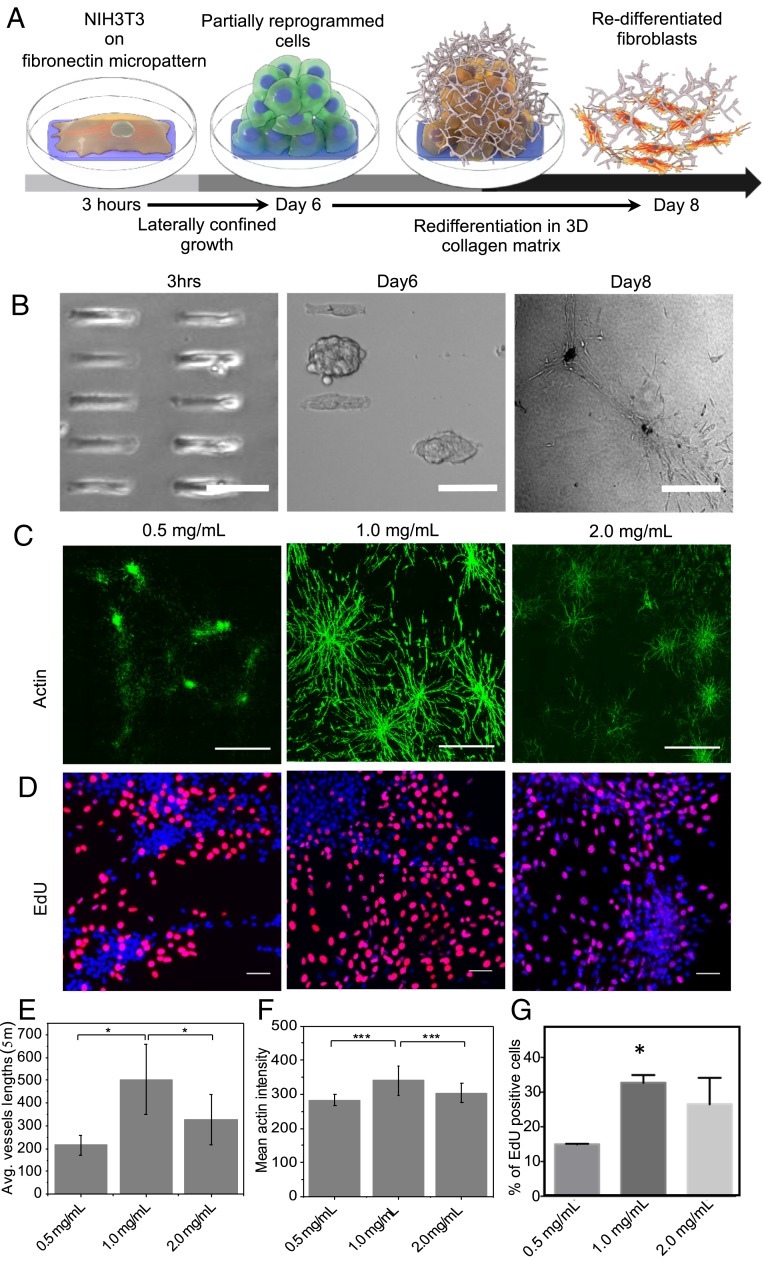
Redifferentiation of fibroblasts from partially reprogrammed spheroids depends on the 3D collagen matrix density. (*A*) Schematic representation of the effect of geometry-driven laterally confined growth of fibroblasts on reprogramming, followed by their redifferentiation within the embedded 3D collagen matrix. (*B*) Phase-contrast images of NIH 3T3 mouse fibroblast cells cultured on micropatterns for up to 6 d, spheroids of PRs, and RFs undergoing sprouting in 3D collagen matrix. (Scale bars, 100 µm.) (*C*) Sprouting efficiencies of cells from 6-d-old spheroids on collagen matrices of varying densities. Cells were stained with phalloidin to label actin. (Scale bars, 500 µm.) (*D*) Proliferation rates of sprouting cells at varying matrix densities using an EdU incorporation assay. (Scale bars, 50 µm.) (*E*–*G*) Sprouting efficiencies, contractility, and proliferation levels at varying matrix densities measured by average (Avg.) vessel length, mean actin intensities, and percentages of EdU-positive cells in each field of view, respectively. For *E* and *G*, *n* > 5 fields of view were randomly measured in each condition. For *F*, number of cells, *n* = 3,304, 4,786, and 1,015 for 0.5, 1, and 2 mg/mL conditions, respectively. Error bars represent ± SD. *P < 0.05; ***P < 0.001. Two-sided Student’s *t* test was used.

In our previous study on mechanically induced nuclear reprogramming in the absence of exogenous biochemical factors, we found that mouse embryonic fibroblasts undergoing laterally confined growth for 6 d on micropatterns started to acquire partial stem-cell-like gene expression (11). These 6-d-old spheroids were embedded in collagen gels and cultured for at least 2 d (Fig. 1*A*). Within a few hours, cells originating from the spheroids progressively invaded the collagen matrix and migrated either individually as unicellular sprouts (single cells) or collectively as complex, capillary-like structures (Fig. 1*B* and *SI Appendix*, Fig. S1). In addition to cell invasion, morphological modifications in the spheroid core itself were detected. While the spheroids initially appeared as a compact structure, subsequent cell migration induced spheroid expansion and led to breaches in the spheroid core. Since cell/substratum adhesion is known to govern cell-sprouting parameters in the 3D matrix (13), we next investigated the influence of physical properties of the 3D matrix, such as its porosity and stiffness (usually combined into a single parameter known as matrix density) on cell-sprouting patterns. We compared cell sprouting in the matrix as a function of matrix density obtained through varying collagen concentration from 0.5 to 2 mg/mL. By using AngioTools analysis (16) to quantify cell sprouting from the spheroids on matrices of varying densities, we found that average sprouting length in these cells showed a biphasic dependence on matrix density (Fig. 1 *C* and *E*). Similar to sprouting length, cell contractility, as measured by actin levels, also showed a similar biphasic trend depending on matrix density (Fig. 1*F*). Addition of cytochalasin D (which depolymerizes actin) and blebbistatin (which inhibit myosin II contractility) led to a significant reduction in average sprouting length, as well as a drop in collagen fiber stiffening (*SI Appendix*, Fig. S1). 1We next investigated the effect of varying matrix density on cell-proliferation rates, using a 5-ethynyl-2′-deoxyuridine (EdU) incorporation assay to quantify DNA synthesis. By measuring the percentage of EdU-positive cells following cumulative incorporation (16 h), we found that proliferation rates were significantly affected by matrix density (Fig. 1 *D* and *G*). Furthermore, we compared the effect of changing collagen density on both redifferentiated fibroblasts (RFs) and fibroblasts clump in collagen gel (FCGs). We embedded the control fibroblasts in three different collagen densities, i.e., 0.5, 1, and 2 mg/mL (*SI Appendix*, Fig. S2*A*). We observed that the sprouting efficiency and contractility (actin mean intensity) was increased with the increasing collagen densities (*SI Appendix*, Fig. S2 *B* and *C*). The proliferation of these FCGs showed a biphasic trend with collagen densities similar to RF cells (*SI Appendix*, Fig. S2*D*). Importantly, the sprouting length of FCG was relatively smaller than the RF condition in all three collagen densities, suggesting that the RF cells could have higher cell-matrix contacts. The 1 mg/mL collagen concentration was therefore selected as the optimal matrix density in all subsequent studies. All these results suggest that an optimal mechanical state of the 3D collagen matrix results in the redifferentiation of PRs into fibroblasts.

### A Shift in Transcription Profiles Is Accompanied by Enhanced Cytoskeletal Gene Expression: RNA-Seq.

**Fig. 2.**
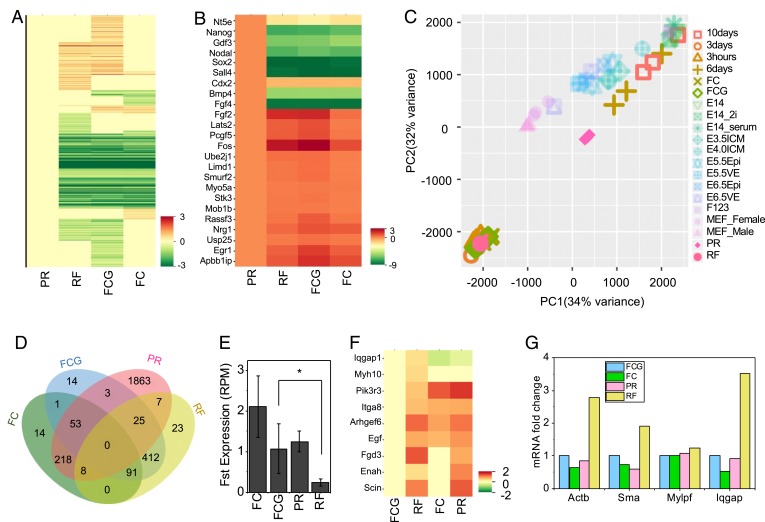
A shift in transcription profile accompanied with enhanced cytoskeletal genes’ expression. (*A*) Heatmap showing fold change (log2) in global transcription profiles between PR, RF, FCG, and FC cells. FDR (adjusted *P* value) < 0.1. (*B*) Heatmap showing log2 fold change in the differential day 6 partially expression of selected genes (compared to the PR sample). FDR (adjusted *P* value) < 0.1. (*C*) PCA showing the shift in cell states through reprogramming and reverting back through redifferentiation. FDR (adjusted *P* value) < 0.01 and |log2 fold change| > 2. (*D*) Venn diagram showing the number of up-regulated genes in 14 (2^*n*^ – 2; *n* is four conditions) comparisons. FDR (adjusted *P* value) < 0.1. (*E*) Bar plot depicting changes in the expression of the representative aging gene Fst. The error bars represent ± SD. *P < 0.1. (*F*) Heatmap showing the log2 fold change in the differential expression of selected cytoskeletal-related genes (compared to FCG). *P* value (not adjusted) < 0.05. (*G*) mRNA levels of selected cytoskeletal genes obtained by qRT-PCR, normalized with respect to FCG.

In order to characterize the gene-expression profiles in RFs and compare them with other control conditions, including PRs, fibroblasts grown in clumps (FCs), and FCGs, RNA-seq experiments were performed. Thousands of genes, including key pluripotency markers Bmp4, Cdx2, Fgf4, Gdf3, Nanog, Nodal, Nt5e, Sall4, and Sox2, were solely up-regulated in the PR cells (Fig. 2 *A* and *B*). When the gene-expression profiles in these four conditions were analyzed, two drastically different cell states were revealed by principal component analysis (PCA) (*Methods* and Fig. 2*C*). PR cells shifted away from the parental fibroblast-like state (FC) to a stem-like state, as a result of lateral confinement (11). Embedding these PRs in the 3D collagen environment led their gene-expression profiles to return to the parental 3T3 fibroblast-like state in RF cells. We observed a difference in gene-expression profiles between cells undergoing reprogramming and original fibroblasts (both cultured on a 3D collagen matrix), which is supported by a Venn diagram showing the number of up-regulated genes in different comparisons (Fig. 2*D*). Based on these comparisons, we identified two groups of genes, which were either selectively overexpressed (23 genes) or down-regulated (53 genes) in RF compared to all of the other conditions. Further, of all of the down-regulated genes, we found that a specific gene, Follistatin (Fst), which is a common marker for aging (17), was expressed at significantly lower levels in RFs than in all other conditions (Fig. 2*E*). Such a significant decrease in Fst expression in RFs indicates the rejuvenation of fibroblasts through the reprogramming process. In order to confirm the difference between the Fst protein level in RF and FCG conditions, we performed Western blotting. Consistent with the RNA-seq result, the Fst protein level was lesser in the RF condition than the FCG condition (*SI Appendix*, Fig. S6 *B* and *C*). Genes up-regulated in RFs formed a molecular interaction network, which was characterized by several connection nodes around proteins such as Rab25, Cdc42bpa, Rhoj, and Iqgap1 that enhance cell migration and cell contractility (*SI Appendix*, Fig. S3*A*) (*Methods*). The expression of selected genes regulating cell contractility was up-regulated in RFs compared to FCGs (Fig. 2*F* and *SI Appendix*, Fig. S3*B*). Further, in agreement with the RNA-seq profile, the increase in messenger RNA (mRNA) levels of selected contractility-related genes was validated by qPCR assay (Fig. 2*G*). These experiments show that PR cells can be redifferentiated into a fibroblast-like (RF) state by embedding them into a 3D collagen matrix, and these cells are characterized by elevated expression of contractility- and rejuvenation-related genes.

### RFs Are Characterized by Enhanced Contractility and Matrix Remodeling.

**Fig. 3.**
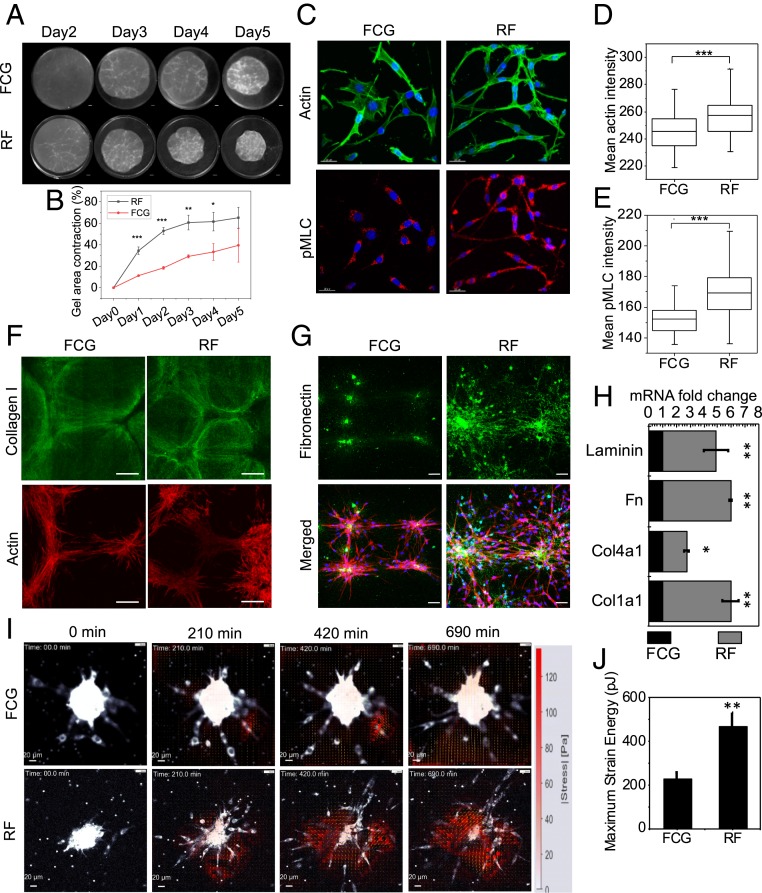
RFs are characterized by enhanced contractility and matrix remodeling. (*A*) Representative images of temporal collagen-gel contraction by matrix-embedded RFs and FCGs. (*B*) Normalized gel area plot representing the gel-contraction efficiencies of these two types of cells. Error bars represent ± SD. The *P* values represent the adjusted *P* values obtained by Bonferroni adjustment methods. *P < 0.05; **P < 0.01; ***P < 0.001. Two-sided Student’s *t* test was used. (*C*) Representative actin and pMLC immunofluorescence micrograph of RFs and FCGs embedded in 1 mg/mL collagen matrix. (Scale bars, 20 µm.) (*D* and *E*) Corresponding box plots for cellular mean intensity of actin and pMLC; *n* = 81 and 67 for FCG and RF conditions, respectively. ***P < 0.001. Two-sided Student’s *t* tests were used. (*F*) Representative fluorescence micrographs of immunostained collagen matrix in these two conditions. Corresponding phalloidin-stained actin images represent cells within the matrix. (Scale bar, 100 μm.) (*G*) Representative immunofluorescence micrographs of extracellular FN deposited on to the matrix in these two conditions. In merged images, the nucleus is labeled in blue, FN in green, and actin in red. (Scale bar, 50 μm.) (*H*) mRNA levels of selected ECM-related genes obtained by qRT-PCR, normalized with respect to FCG. Error bars represent ± SD. *P < 0.05; **P < 0.01. Two-sided Student’s *t* test was used. (*I*) Representative temporal traction force maps quantifying forces exerted on the matrix during sprouting of these two cell types. (*J*) Corresponding maximum strain energy plots during sprouting of these two cell types. Error bars represent ± SD. **P < 0.01. Two-sided Student’s *t* test was used.

In order to characterize these RFs in vitro, we compared the gel-contraction abilities of cells derived from PR spheroids or from FCs, both of which were equally embedded in the 3D collagen matrix. Representative images and quantitative analysis of gel-area reduction revealed that the amount of collagen-gel contraction by the RFs was higher compared to FCGs (Fig. 3*A*). Fibroblasts exhibit contractile actin bundles (18), and, therefore, we compared actin and phosphorylated myosin light chain (pMLC) global intensities in these two cell types embedded in the collagen matrix. In agreement with the RNA-seq results, Fig. 3 *C*–*E* and *SI Appendix*, Fig. S4 clearly show that the RFs exhibited enhanced actomyosin contractility compared to control fibroblasts (FCGs). Fibroblasts are known to exert mechanical forces on the ECM surrounding them. Hence, we studied fibroblast-induced reorganization of the matrix by visualizing immunostained collagen fibers and qualitatively evaluating the effect of different inhibitors on collagen-fiber remodeling. Both RFs and FCGs embedded in fibrillary collagen matrix were able to remodel collagen fibrils into thicker bundles (Fig. 3*F*). Remodeling of collagen fibrils into thick bundles was observed within the fibroblast-populated collagen gel. We observed that collagen fibrils rearranged thicker around RFs compared to control FCG samples. In addition, to check the expression level of collagen–cross-linking molecules Lox in RF and FCG cells, we plotted the Lox mRNA level from the RNA-seq data. RF cells showed higher Lox expression compared to FCG cells (*SI Appendix*, Fig. S6*D*). This result suggests the enhanced remodeling properties of RF cells compared to FCG. However, collagen fibrils around RFs exhibited very less or no remodeling in samples treated with 25 µM Y-27632 (a RhoA-kinase inhibitor) and 4 µM cytochalasin D (inhibitor of actin polymerization) (*SI Appendix*, Fig. S1). Matrix assembly and remodeling are usually promoted by ECM glycoproteins that bind to cell-surface receptors, such as fibronectin (FN) dimers binding to integrins. Fibroblasts deposit FN to the matrix along the way of migration. Immunostaining experiments showed that both the fibroblasts migrated within the 3D collagen matrix; however, RFs deposited more FN along their migration trails compared to control FCGs (Fig. 3*G* and *SI Appendix*, Fig. S5). In addition, these RFs also expressed several other ECM-related genes, including Lama1 (laminin, alpha 1), Fn1 (fibronectin 1), Col4a1, and Col1a1 at higher levels than in controls, as quantified by qPCR assay (Fig. 3*H*). Further, we performed Western blotting analysis to confirm the difference between the Fst protein level in the RF and FCG conditions. Consistent with the RNA-seq result, the Fst protein level was lesser in the RF condition than the FCG condition (*SI Appendix*, Fig. S6 *A* and *C*). Fibroblasts embedded in the 3D ECM are mechanically supported by the ECM and, in turn, exert forces onto the ECM through cell–ECM contacts (1, 19). A temporal quantitative measurement of the contractile forces exerted by these two fibroblast types was done by using 3D traction force microscopy (TFM) during fibroblast sprouting (20). A color map based on the measurements indicated that RFs exerted comparatively higher traction stress during the initial 12 h of the sprouting phase (Fig. 3*I*). Vector arrows are indicated in the direction of force at each small window. During sprouting of cells from spheroids, the peak strain energy exerted by cells varied between spheroids, with a maximum energy of 450 and 220 pJ exerted by RFs and FCGs, respectively (Fig. 3*J* and *SI Appendix*, Fig. S7*A*). Considering the nonlinear properties of the collagen, we also measured the traction force of FCG and RF cells seeded on a two-dimensional (2D) FN-coated soft polydimethylsiloxane (PDMS) substrate (described in *Methods*). Consistent with the 3D TFM results, we observed that the RF cells showed higher 2D traction compared to the FCG cells (*SI Appendix*, Fig. S7 *B* and *C*). All together, these results support that augmented contractility and enhanced matrix remodeling are characteristics of the RFs.

### Rejuvenation through Redifferentiation of Partially Reprogrammed Fibroblasts Ameliorates Age-Associated Phenotypes.

**Fig. 4.**
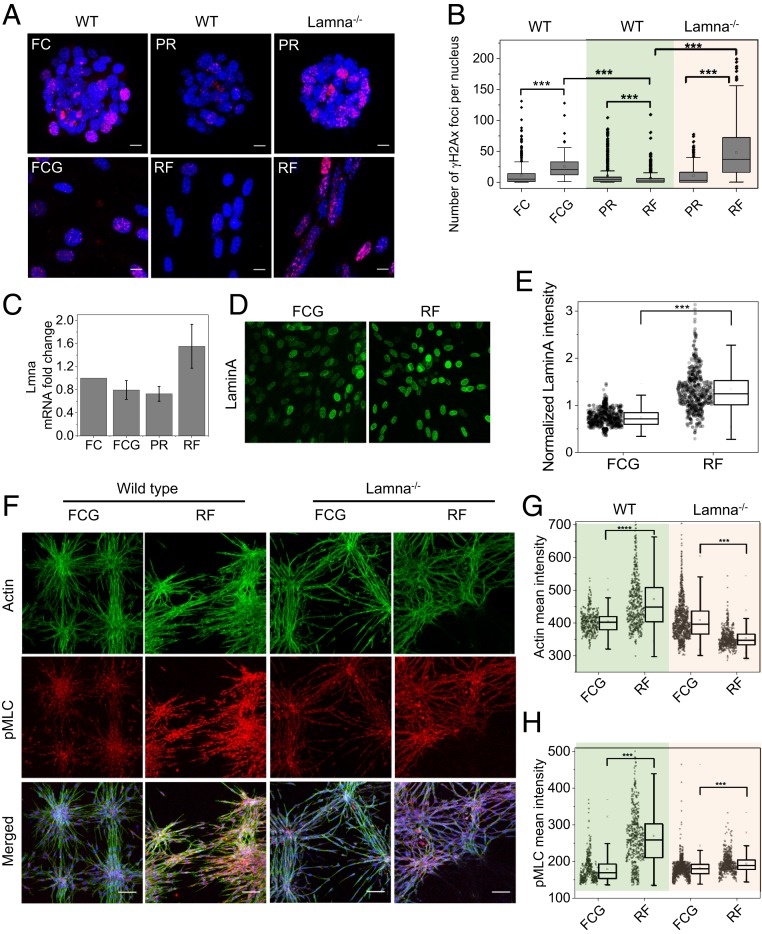
Recovering from age-associated hallmarks DNA damage by partial reprogramming and LaminA dependency. (*A*) Representative gH2AX immunofluorescence micrographs of cell nuclei in different conditions: with and without gel and in both wild-type 3T3 (WT) and Lmna knockout 3T3 cell lines (Lamna^−/−^). (Scale bars, 10 µm.) (*B*) Corresponding box plots of gH2AX foci per nucleus. *n* = 549, 93, 522, 473, 323, and 545 for respective conditions. ***P < 0.001. Two-sided Student’s *t* tests were used. (*C*) mRNA levels of Lmna in different conditions obtained by qRT-PCR normalized with respect to NIH 3T3 clumps on patterns; error bars represent SD. (*D*) Representative LaminA immunofluorescence micrographs of the nuclei in fibroblasts and 3T3 cells embedded in the collagen matrix. (Magnification: 200×.) (*E*) Corresponding box plot for normalized nuclear LaminA intensities. *n* = 633 and 554 for FCG and RF conditions, respectively. ***P < 0.001. Two-sided Student’s *t* tests were used. (*F*) Representative actin and pMLC immunofluorescence micrographs of fibroblasts and 3T3 cells embedded in collagen matrix in both WT and Lmna^−/−^ conditions. In merged images, nucleus is in blue. (Scale bars, 50 µm.) (*G* and *H*) Corresponding box plots for cellular mean intensity of actin and pMLC; *n* = 400, 558, 1,114, and 619 for the respective conditions. ***P < 0.001; ****P < 0.0001. Two-sided Student’s *t* tests were used.

In order to investigate whether aging-associated phenotypes improve following rejuvenation, we next analyzed the level of DNA damage in these cells. Interestingly, the number of foci containing histone gH2AX, a marker of nuclear DNA double-strand breaks associated with aging (21), were significantly reduced in RFs compared to FCGs (Fig. 4 *A* and *B*). Lateral confinement induced PR cells to accumulate significantly fewer gH2AX foci compared to FCs. Sprouting of FCs induced by constriction of pores in the 3D collagen matrix resulted in an increase in gH2AX foci, whereas the change in the number of gH2AX foci in RFs was decreased compared to that in PR cells (Fig. 4 *A* and *B*). Cell migration through constricting pores can lead to accumulation of DNA damage, which is dependent on its nuclear lamina levels (22). By using qPCR to quantify Lmna gene regulation, we found a decrease in Lmna mRNA levels in sprouted cells derived from FCs compared to FCGs (Fig. 4*C*). Interestingly, Lmna mRNA levels increased during redifferentiation in the RFs. In agreement with the qPCR data, immunofluorescence data showed a significant increase in LaminA levels in the RF compared to the FCG condition (Figs. 4 *D* and *E* and *SI Appendix*, Fig. S8). In addition, an increase in the number of gH2AX foci in Lamna^−/−^ RFs suggests that higher LaminA levels in wild-type RFs may act to shield their nuclei from accumulating DNA damage during migration through constricted pores in the collagen matrix (Fig. 4 *A* and *B*). The nuclear lamina can regulate cellular contractility, and vice versa (23). Therefore, we next investigated the relationship between LaminA changes in rejuvenated cells and contractility. We found that the actin level was significantly increased in RFs compared to FCGs, yet when lmna^−/−^ cells were used, RFs exhibited decreased actin compared to FCGs (Fig. 4 *F* and *G*). However, we observed an increase in pMLC levels in Lmna^−/−^ RFs, although not as high as in wild-type RFs (Fig. 4 *F* and *H*). Collectively, these results demonstrate that short-term, in vitro induction of fibroblast reprogramming through lateral confinement of 3T3 cells, followed by their subsequent redifferentiation can ameliorate phenotypes associated with physiological aging (e.g., accumulation of DNA damage and nuclear-envelope defects) by increasing LaminA levels in their nuclei.

### Chromatin Poised States in PRs.

**Fig. 5.**
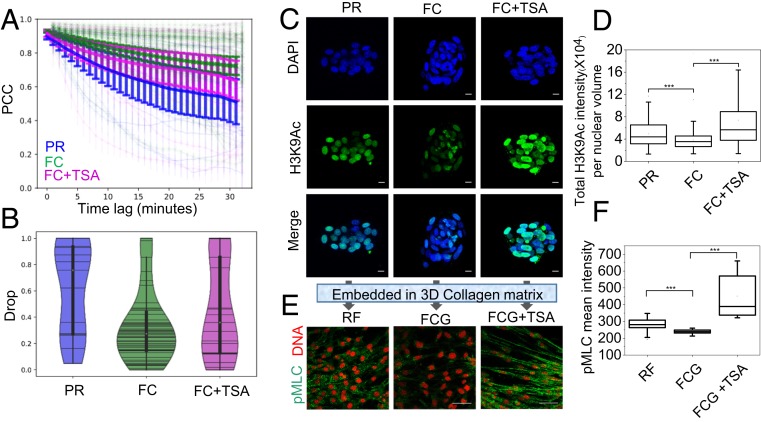
Chromatin poised state in PRs. (*A*) PCC curve of projected nuclear area as a function of time with mean and CI of mean (as error bar). *n* = 38, 138, and 122 for PR, FC, and FC+TSA conditions, respectively. (*B*) Drop rate obtained by fitting the nuclear area PCC curves of *A*, indicating the rapid change of nuclear morphology and DNA organizations in PR and FC+TSA conditions. The procedure of analysis of *A* and *B* is described in *Methods*. (*C*) Representative H3K9Ac immunofluorescence micrographs of the nuclei in Day6, 3T3 clump, and 3T3 clump+ TSA-treated cells without gel conditions. (Scale bars, 10 µm.) (*D*) Corresponding box plots of total H3K9Ac intensities per nuclear volume. *n* = 383, 788, and 903 for PR, FC, and FC+TSA, respectively. ***P < 0.001. Two-sided Student’s *t* tests were used. (*E*) Representative pMLC immunofluorescence micrographs of the above-mentioned cell types embedded in collagen matrix for 2 d. Nucleus is labeled in red. (Scale bars, 50 µm.) (*F*) Corresponding box plots of mean pMLC intensities in these three conditions. Numbers of fields of view used for analysis are *n* = 23, 58, and 15 for RF, FCG, and FCG+TSA, respectively. ***P < 0.001. Two-sided Student’s *t* tests were used.

The pluripotent genome is characterized by unique epigenetic features and a decondensed chromatin conformation (24). Therefore, we hypothesized that rejuvenation of fibroblasts may be a result of the chromatin poised state in the PR cells. We first examined the nuclear dynamics in PR cells and FCs and in FCs treated with Trichostatin A (TSA), a specific inhibitor of histone deacetylase (HDAC). As expected, time-lapse laser-scanning confocal microscopy of Hoechst 33342-stained nuclei showed an increase in nuclear dynamics in PRs and TSA-treated FCs, compared to control FCs (Fig. 5 *A* and *B*). This also correlated with low levels of LaminA in the PR nucleus compared to RF (Fig. 4*C* and *SI Appendix*, Fig. S8). Treatment with TSA increased the nuclear levels of H3K9ac, a marker of chromatin decondensation. In agreement with the nuclear dynamics results, immunofluorescence experiments clearly revealed a significant increase in H3K9ac levels in the PRs and TSA-treated FCs, compared to untreated FCs (Fig. 5 *C* and *D* and *SI Appendix*, Fig. S9). In order to explore levels of actomyosin contractility, we embedded TSA-treated FCs in a 3D collagen matrix and assayed the level of pMLC in sprouted cells as a measure of contractility. Interestingly, we found a higher level of pMLC in the TSA-treated FCGs compared to untreated FCGs and RFs (Fig. 5 *E* and *F* and *SI Appendix*, Fig. S10). These results suggest that during lateral confinement-induced reprogramming, cells undergo chromatin decondensation that may enhance the activity of target genes in response to matrix cues, promoting cellular processes essential for rejuvenation.

### Validation of Fibroblast Rejuvenation in Human Fibroblasts.

**Fig. 6.**
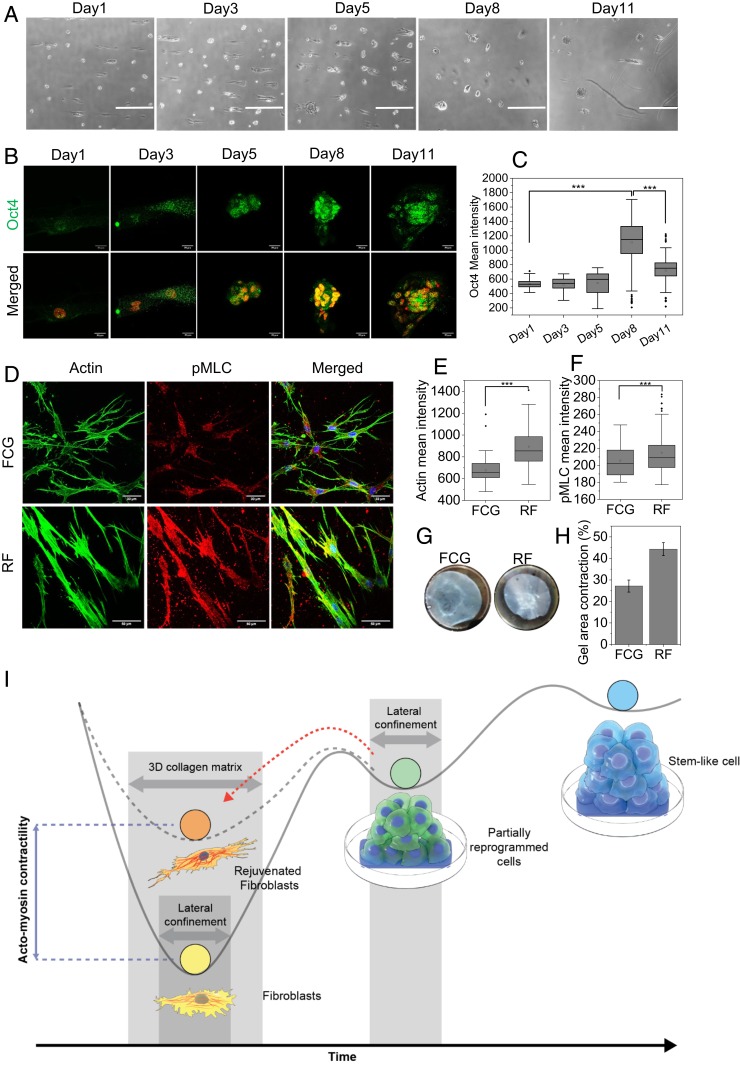
Rejuvenation of human aged fibroblasts. (*A*) Phase-contrast images of human primary aged skin fibroblast (GM08401) cells cultured on micropatterns for up to 11 d. (Scale bars, 500 µm.) (*B*) Representative micrograph of GM08401 spheroids at different days immunostained with Oct4. (Scale bars, 20 µm.) (*C*) Nuclear Oct4 mean intensity plot of spheroids at different days. ***P < 0.001. (*D*) Representative actin and pMLC immunofluorescence micrograph of RF and FCG of GM08401 embedded in 1 mg/mL collagen matrix. (Scale bars, 33 µm [*Upper*] and 50 µm [*Lower*].) (*E* and *F*) Corresponding box plots for cellular mean intensity of actin and pMLC in FCG and RF conditions, respectively. ***P < 0.001. Two-sided Student’s *t* tests were used. (*G*) Representative images of collagen-gel contraction by matrix embedded with GM8401 RF and FCG. (*H*) Normalized gel area plot representing the gel-contraction efficiencies of these two types of cells. (*I*) Schematic summary of the induction of reprogramming by laterally confined growth of cells and followed by redifferentiation into RFs in 3D collagen matrix.

In order to validate the rejuvenation results in the human fibroblast model, we used the similar experimental approach to rejuvenate aged and young human fibroblasts. As an aged and young fibroblast model, we used primary skin fibroblasts obtained from an aged donor (age 75) (GM08401, Coriell Institute) and human foreskin fibroblast cell line from newborn (BJ cells), respectively. GM08401 cells were grown on laterally confined conditions on a specific FN micropattern (area 9,000 μm^2^ with aspect ratio [AR] 1:4) for 11 d until the spheroid formation (Fig. 6*A*). The partial reprogramming of the GM08401 was confirmed through the Oct4 immunostaining (Fig. 6 *B* and *C*). Further, we redifferentiated these partially reprogrammed GM08401 cells similarly by embedding them on a 1 mg/mL collagen matrix for 3 d until they sprouted out in the collagen gel. Similarly, for control, we formed the FCs of aged cells and embedded in the collagen gel for FCGs. In order to compare the contractility between the RFs and FCGs of aged cells, we measured the pMLC and actin levels by immunofluorescence. Interestingly, we also observed the higher level of actin and pMLC in the RF cells compared to the FCG cells (Fig. 6 *D*–*F*). In addition, to show the gel-contraction potential of these two types of fibroblasts, we performed the gel-contraction assay similarly as described before. In agreement with the actomyosin contractility, we also observed that gel with RF cells contracted 44%, whereas gel with FCG cells contracted only 27% after 5 d of culture (Fig. 6 *G* and *H*). These results suggest that by using a similar approach, the aged cells can also be rejuvenated with higher actomyosin contractility. To further validate the rejuvenation process in the young human fibroblasts model, BJ cells were grown on laterally confined conditions on a specific FN micropattern for 10 d. The partial reprogramming was confirmed through the increased level of alkaline phosphatase and Oct4 mRNA and protein expression (*SI Appendix*, Fig. S11 *A*–*D*). Further, we redifferentiated these partially reprogrammed BJ cells similarly by embedding them on a 1 mg/mL collagen matrix. These were then allowed to sprout and grow for 48 h. The redifferentiated cells (RFs) showed higher contractility in terms of pMLC level as compared to the control BJ fibroblasts (FCGs) (*SI Appendix*, Fig. S11 *E* and *F*). Further, in agreement with the NIH 3T3 cells, we also observed increased LaminA and decreased gH2AX levels in the redifferentiated BJ fibroblasts compared to control fibroblasts (*SI Appendix*, Fig. S11 *G*–*I*). In order to compare between the aged, rejuvenated fibroblasts (RFs) derived from aged fibroblasts and young fibroblasts, we used GM08401 (age 75; Coriell Institute) and GM01652 (age 11; Coriell Institute) as a more appropriate aged and young human primary skin fibroblasts model, respectively. The RFs were obtained from the aged primary skin fibroblast (GM08401, age 75; Coriell Institute) similar to as mentioned previously in the manuscript. For control young and aged fibroblast conditions, similarly, we formed the FCs of GM01652 and GM08401cells and embedded them in 1 mg/mL collagen matrix for respective FCGs. A prominent characteristic of dermal fibroblasts in aged skin is reduced size, with decreased elongation and a more rounded, collapsed morphology. Conversely, young and healthy fibroblasts normally attach to the ECM strongly and thereby achieve stretched, elongated morphology (5, 25). Importantly, we show that the RFs exhibit increased elongated morphology compared to their more rounded parental aged fibroblasts (GM08401), but similar to the control young fibroblasts (GM001652) (*SI Appendix*, Fig. S12 *A* and *B*). The cell-area analysis in 3D collagen matrix showed significant increased cell area upon rejuvenation of aged cells, and the cell area of these RFs was similar to control young fibroblasts (FCGs; GM01652). Interestingly, we also observed the higher level of pMLC in the RF cells compared to the aged fibroblasts (GM08401), but similar to control young fibroblasts (GM01652) (*SI Appendix*, Fig. S12 *A* and *C*). These results suggest that aged fibroblasts, upon rejuvenation, regain some of the characteristics of young fibroblasts (cell area and pMLC levels). Collectively, these results show similar types of rejuvenation characteristics of fibroblasts originated from either partially reprogrammed human (BJ) or mouse (NIH 3T3) fibroblasts or aged fibroblasts (GM08401). A schematic summary of the nuclear reprogramming processes induced by laterally confined growth of fibroblasts and their subsequent rejuvenation during redifferentiation within the 3D collagen matrix is shown in Fig. 6*I*.

## Discussion

Somatic cell nuclear transfer and iPSCs have been used in the cellular-rejuvenation process in many studies, for example, rejuvenation of aged fibroblasts, neurons, cardiac myocytes, T cells, macrophages, and skin cells (8, 26). While all these methodologies have enormous applications, their clinical use is limited by disadvantages such as lower efficiency and increased risk of oncogenic transformations in cells due to genomic mutations acquired during the dedifferentiation process (10, 27). Therefore, in recent years, several approaches, including environmental (heterochronic parabiosis), genetic (down-regulation of nuclear factor NF-κB signaling), and pharmacological methods (mammalian target of rapamycin inhibition by rapamycin can extend the life span of mice), have been applied to rejuvenate cells without attaining complete dedifferentiation (28).

Despite the existence of several nongenetic approaches for dedifferentiation that involve the use of small molecules or mixtures of transcription factors, physical routes of reprogramming and their potential to overcome the above-mentioned limitations of dedifferentiation have not been clearly demonstrated. In addition, the rejuvenation of such physically dedifferentiated cells by their redifferentiation into more active cellular states have not been explored. In our recent study, we showed that the laterally confined growth of fibroblasts on FN micropatterns induces their reprogramming and confers on them embryonic-stem-cell (ES)-like characteristics (11). Along with the potential to restore stem-cell-like properties, this mechanical mode of reprogramming also opens up avenues for potential implication in the field of rejuvenation (29).

In this paper, we used these PR cells (generated by laterally confined growth of fibroblasts) with naive ES-like expression profiles and redifferentiated them into fibroblasts. This approach also highlights the advantage of decoupling rejuvenation from complete dedifferentiation. Redifferentiation of stem cells into a specific lineage can be augmented by the mechanical properties of the tissue microenvironment. Here, we defined an optimal 3D mechanical microenvironment for the redifferentiation of partially reprogrammed spheroids, by controlling stiffness and pore size of the collagen-I matrix (12, 13). The mechanical properties of the 3D collagen matrix, such as its stiffness, may act as regulatory checkpoints during the rejuvenation of fibroblasts on the matrix. These results suggest that optimal matrix stiffness and pore size (mimicking the architecture of the physiological tissue) induce efficient redifferentiation of these PRs into fibroblasts.

Recent evidences from electron-microscopy and electron-spectroscopy imaging of chromatin structures indicated that undifferentiated ES cells and iPSCs exhibit an open chromatin state compared to differentiated cells (24, 30). In agreement with these observations, fibroblasts that were partially reprogrammed under laterally confined growth conditions showed higher nuclear dynamics as well as enriched active histone marks (H3K9Ac), suggesting a more open chromatin structure compared to control fibroblasts. The open chromatin state is transcriptionally silent, but poised for activation, as its bivalent histone domains can be rapidly activated (through the loss of H3K27me3) when differentiation is induced. During redifferentiation, PR cells migrate through a small mesh size of 2 to 4 μm, enabling their open chromatin to be exposed to matrix signals. The cascade of collagen-I matrix-dependent downstream-signaling pathways in this highly open chromatin state may enable relatively increased transcription of their target genes, leading to rejuvenation. Transcriptional analysis showed an up-regulation of laminA and other contractility- and rejuvenation-related markers in RFs compared to control cells, suggesting that RFs evolve from normal fibroblasts through reprogramming. In addition, treatment of fibroblasts with agents that promote chromatin decondensation, such as the HDAC inhibitor TSA resulted in chromatin that was more poised for activation, as these cells showed increased contractility upon exposure to ECM-related cues.

The mechanical reprogramming of fibroblasts, followed by their redifferentiation into RFs in an optimized 3D collagen matrix, made these cells more contractile and more efficient at synthesizing matrix components including laminin, FN, and collagen-IV. Moreover, the RFs obtained through this approach exhibited a decrease in DNA damage. The RFs derived from this method precisely aligned into tissue architectures, suggesting their potential application as clinical implants in tissue-engineering and regenerative medicine.

## Methods

### Partial Reprogramming of Fibroblasts and Redifferentiation.

NIH 3T3 mouse embryonic fibroblasts were cultured on FN micropatterns and grown under laterally confined conditions as described (11). Briefly, rectangular (AR 1:5) micropatterns measuring 1,800 μm^2^ were created on uncoated Ibidi dishes (81151) by stamping of FN (catalog no. F1141; Sigma)-coated PDMS micropillars fabricated by soft lithography. This was followed by surface passivation of the micropatterned dish with 0.2% pluronic acid (Sigma catalog no. P2443) for 10 min. NIH 3T3 cells were expanded in high-glucose Dulbecco’s modified Eagle medium (DMEM) (Gibco) + 10% (volume [vol]/vol) FBS (Gibco) and 1% penicillin–streptomycin (Gibco). For partial reprogramming, NIH 3T3 cells were seeded on an FN-micropatterned dish (rectangles spaced 150 μm apart) at a concentration of ∼7,000 cells per dish, to reach a density of one cell per FN island. Single cells were grown in under laterally confined conditions for 6 d in the above-mentioned culture medium, with a fresh medium replenishment on every alternate day unless otherwise stated. In control 3T3 clump conditions (FCs), similar spheroid size and cell density (compared to 6-d partially reprogrammed spheroids) was achieved by seeding NIH 3T3 cells on differently spaced micropattern dishes (500 μm) at a concentration of ∼80,000 cells per dish and growing them overnight. For redifferentiation, spheroids or cells obtained from trypsinized spheroid were embedded in 3D rat tail collagen-I gel of varying concentration (0.5 to 2 mg/mL), according to the manufacturer’s protocol (Thermo Fisher). In such a 3D collagen matrix, cells were cultured for 48 h in the above-mentioned medium for most of the rejuvenation assays unless otherwise stated. In order to partially reprogram human foreskin fibroblasts (BJ cells), cells were grown on laterally confined conditions similar to NIH 3T3 cells on a specific FN micropattern (area 3,364 μm^2^, AR 1:4) for 10 d in high-glucose DMEM (Gibco) + 10% (vol/vol) fetal bovine serum (FBS) (Gibco) and 1% penicillin–streptomycin (Gibco). Similar to redifferentiation of NIH 3T3 cells, these partially reprogrammed BJ cells were further redifferentiated by embedding them on a 1 mg/mL collagen matrix. The aged and young human primary skin fibroblasts were obtained from an aged donor (age 75) (GM08401; Coriell Institute) and a young donor (age 11) (GM01652; Coriell Institute), respectively. GM08401 cells were cultured and grown on laterally confined conditions on a specific FN micropattern (area 9,000 μm^2^ with AR 1:4) in a 1:1 high-glucose DMEM (Gibco) and minimal essential medium (Gibco) supplemented with 15% (vol/vol) heat-inactivated FBS (Gibco) and 1% penicillin–streptomycin (Gibco). For respective young and aged fibroblasts, FCs were obtained on FN micropattern (area 9,000 μm^2^ with AR 1:4) and followed by embedding them in 1 mg/mL collagen matrix for their respective FCGs.

### Cell-Proliferation Assay.

The percentage of cells (cultured in collagen-I gel for 24 h) in the S phase was evaluated by using an in situ cell-proliferation kit (Click-iT EdU Alexa Fluor 555 Imaging Kit, Thermo Fisher Scientific) that quantified the incorporation of EdU into cellular DNA. As per the manufacturer’s instructions, cells in 3D collagen-I matrix were allowed to incorporate 10 μM EdU for 16 h. After EdU incubation, cells were fixed with 4% paraformaldehyde and permeabilized with 0.5% Triton for 20 min. Following this, cells were incubated with 0.5 mL of Click-iT reaction mixture for 30 to 35 min at room temperature and then washed with phosphate-buffered saline (PBS). Cell nuclei were counterstained with DAPI.

### RNA-Seq Sample Preparation and Analysis.

Total RNA was isolated from cells grown on patterns for varying times by using the RNeasy Plus Micro Kit (Qiagen). Cells grown in 3D collagen-I gel were treated with collagenase for 15 min prior to RNA isolation. The preparation of the mRNA library (Illumina Stranded) and sequencing on a HiSeq 2000 platform were performed at the Genome Institute Singapore. In summary, we had four conditions: FCs (3T3 clumps grown overnight on micropatterns without gel), FCGs (3T3 clumps grown in collagen-I gel for 48 h), PRs (partially reprogrammed spheroids without gel), and RFs (6-d samples grown in collagen-I gel for 48 h); each condition had three biological replicates and four technical replicates (run on four different lanes). Reads were aligned to *Mus musculus* GRCm38.p6 soft-masked genomic DNA (with GenBank Assembly ID GCA_000001635.8, downloaded from Ensembl) using the TopHat sequence-alignment tool. The annotation file (gene transfer format) used for TopHat sequence alignment was downloaded from Ensembl (for GRCm38.p6 assembly) (31). Default parameters were used in TopHat (Version 2.1.1) (32). After alignment, four technical replicates for each biological sample (accepted_hits.bam files from TopHat output) were combined together for downstream analysis. Cufflinks (Version 2.2.1) software was used to assemble the transcripts and obtain the number of reads for each transcript (33). The number of reads for transcripts from the same gene were summed to get the count number (reads per million). Count numbers for all expressed genes were used in differential expression analysis using DESeq2 (Version 1.20.0) (34). Differentially expressed genes had adjusted *P* values (Benjamini–Hochberg) below a 0.1 false discovery rate (FDR) (*P* value thresholds used in other analyses are described in respective figure legends). All of the public RNA-seq datasets were downloaded from the National Center for Biotechnology Information Sequence Read Archive (NCBI-SRA) database, and detail accession IDs are listed in *SI Appendix*, Table S3. The RNA-seq datasets generated in this study (FC, FCG, PR, and RF conditions) are available in Dataset S1. The unit is reads per million mapped reads.

### qRT-PCR.

To investigate selected gene expressions in the four conditions mentioned above, qRT-PCR was performed. By using an iScript complementary DNA (cDNA) synthesis kit (Bio-Rad), cDNA was synthesized from total RNA that was isolated as mentioned previously. Real-time PCR detection was performed by using an SsoFast qPCR kit (Bio-Rad) for 40 cycles in a Bio-Rad CFX96. The relative fold changes in the gene levels were obtained from qRT-PCR data, by using ΔΔCt methods with respect to glyceraldehyde 3-phosphate dehydrogenase (GAPDH) levels. The primer sequences for the selected genes are listed in *SI Appendix*, Table S1.

### Collagen-I Contraction Assay.

To initiate the gel-contraction assay, an equal number of cells obtained from trypsinized 6-d spheroids or clumps were mixed with collagen-I solution, cast in new, uncoated Ibidi dishes (81151), and cultured for 5 d. As a measure of fibroblast contractility, collagen-I gel contraction by the fibroblasts was performed by measuring the fractional decrease in gel area with time.

### TFM.

Quantitative measurement of the force exerted by cells embedded within a collagen matrix was measured by 3D TFM (20, 35). Briefly, cells in PR spheroids or FCs were fluorescently labeled with Cytotracker red (Thermo Fisher) before they were embedded within the collagen matrix. Fluorescent beads of 4-µm size were mixed with 1 mg/mL collagen-I gel composition at a concentration of 60,000 to 80,000 beads per 400 µL of gel solution. Subsequently, fluorescently labeled spheroids or clumps were mixed with collagen solution and cast in uncoated Ibidi dishes for polymerization at 37 °C in a CO_2_ incubator. Once cells started to migrate within 4 h of incubation, the bead and cell displacements were tracked for 18 h under a confocal microscope, at an interval of 30 min. Traction forces were analyzed by using Fourier transform traction cytometry, according to the method discussed in Kovari et al. (20). Particle-displacement rates were calculated by using particle-tracking velocimetry, based on algorithms. Particle displacements were interpolated into a regularized grid corresponding to the approximate substrate displacement. Particle-tracking errors were eliminated by using the filtering procedure. From the displacement field, the traction maps were quantified by using MATLAB. For 2D traction force measurement, we prepared the soft PDMS substrate for traction force as described by Das et al. (36). Briefly, we prepared the ultrasoft PDMS substrate by mixing base (Sylgard 184, Dow Corning Corp.) to cross-linker in a 65:1 ratio (weight/weight). Fluoro-spheres of diameter 46 ± 6 nm (Molecular Probes, Thermo Fisher) were added to the PDMS mixture, followed by careful stirring for at least 15 min. The PDMS–bead mixture was spread on to Ibidi glass-bottom dishes. The coated dishes were then kept at 25 °C for a period slightly more than 1 h. Subsequently, the PDMS-coated dishes were incubated at 50 °C for 4 h for desired cross-linking. These were then treated with oxygen plasma and coated with FN at a concentration of 10 μg/mL for 2 h at 37 °C. RFs and FCGs were isolated from the collagen gel by partial treatment of collagenase and trypsinization. The single cell of RF and FCG were seeded on the TFM substrate at a low density and incubated overnight. Calculation of the displacement field from the fluorescent images of the bead-embedded substrate with and without cells were performed. From the displacement field, the traction maps were quantified by using MATLAB.

### Immunostaining.

Cells embedded in collagen-I gel were fixed with 4% paraformaldehyde (Sigma) in PBS buffer (pH 7.4) for 25 min, followed by washing with PBS + 100 mM glycine buffer (5 min × 3). Cells were permeabilized by using 0.5% Triton (Sigma-Aldrich) in PBS for 20 min, followed by washing with PBS–glycine buffer (5 min × 3). After that, cells were blocked with 10% goat serum (Thermo Fisher Scientific) in immunofluorescence (IF) wash buffer (PBS + 0.2% Triton + 0.2% Tween 20) for 3 h at room temperature. Next, cells were incubated overnight with different primary antibodies diluted in blocking buffer, followed by washing with IF wash buffer (15 min × 3). Cells were then incubated with corresponding fluorescent-labeled secondary antibodies diluted in 5% goat serum in IF wash buffer for 3 h at room temperature. The details of all primary and secondary antibodies and corresponding working dilutions are listed in *SI Appendix*, Table S2. Cell nuclei were stained with NucBlue Live Ready Probes (Molecular Probes; Thermo Fisher Scientific) in PBS for 10 min at room temperature, and filamentous actin was labeled by using phalloidin Alexa Fluor 488 or 568 (1:100; Molecular Probes; Thermo Fisher Scientific) for 45 min.

### Image Acquisition and Analysis.

Fluorescent images of 3D spheroids and cells embedded in 3D collagen-I gel were acquired by using a Nikon A1R laser-scanning confocal microscope (Nikon Instruments Inc.) at either 20× magnification (Plan Apo 20× extra long working distance, numerical aperture [NA] 0.8) or 63× magnification (1.25-NA oil objective) with identical acquisition settings. In the Z dimension, each spheroid and 3D collagen-I gel was scanned up to a depth of 50 µm, with a step size of 1 to 5 µm. Confocal images of either 512 × 512 or 1,024 × 1,024 pixels were obtained with an XY optical resolution of 0.42 or 0.21 µm, respectively. Time-lapse imaging was done in confocal mode for up to 60 min and 18 h with 60-s and 30-min time intervals, respectively. Bright-field images were acquired by using the EVOS FL Cell Imaging System (Thermo Fisher Scientific). For gel-contraction assay, images of collagen gel at different days of cell growth were acquired with a mobile camera at a fixed magnification. The fluorescence intensity was measured for each protein in its respective channel, and the number of gH2Ax foci per nucleus was determined by using custom-written code in Fiji (NI) MATLAB (MathWorks) and IMARIS8. The sprouting length of each spheroid embedded in 3D collagen matrix was measured from the large-field fluorescent micrograph of actin by using AngioTools software.

Nuclear dynamics were analyzed from the decorrelation of the nuclear images in time as described (37). Time-lapse live imaging of nucleus stained with Hoechst 33342 (Thermo Fisher Scientific) was done in confocal mode with time intervals of 1 min for up to 32 min in PR, FC, and FC + TSA conditions. One Pearson correlation coefficient (PCC) value was calculated from two lists of pixel intensity of the same nucleus captured in different time points with a certain time lag. For each cell, one PCC curve was drawn which connected all PCCs (as *y*) with the increasing time lags (as *x*), as represented in dim color in Fig. 5*A*. The mean PCC curves for all cells in each condition were drawn in bright color in Fig. 5*A*. The mean PCC curves in each condition were fitted by equation *y* = (1 − α) + α exp(−*t*/τ) − η, where *y* refers to PCC value, *t* is time lags, fitting parameter α is drop rate, τ is time constant, and η is noise.

### Statistical Analysis.

All data are expressed as mean ± SD or ± SEM as noted in figure legends. For box plots, box limit represents the 25th to 75th percentile and whiskers 1.5× interquartile range. Each experiment was repeated at least three times. We evaluated statistical significance of mean with the Student’s unpaired two-tailed *t* test, performed between sample of interest and corresponding control. *P < 0.05; **P < 0.01; ***P < 0.001.

### Data Availability Statements.

All relevant data are within the manuscript and its *SI Appendix* files. The RNA-seq datasets generated in this study can be found in Dataset S1. The NCBI-SRA accession IDs of public RNA-seq datasets used in this study are listed in *SI Appendix*, Table S3.

## Supplementary Material

Supplementary File

Supplementary File
