# Supplementary material for: Fibroblast rejuvenation by mechanical reprogramming and redifferentiation
Source: Proc Natl Acad Sci U S A. 2020 Apr 29;117(19):10131–41. doi: 10.1073/pnas.1911497117 (PMC7229653; doi:10.1073/pnas.1911497117)
Supplement: Supplementary File [file pnas.1911497117.sapp.pdf]

SUPPLEMENTARY FIGURES

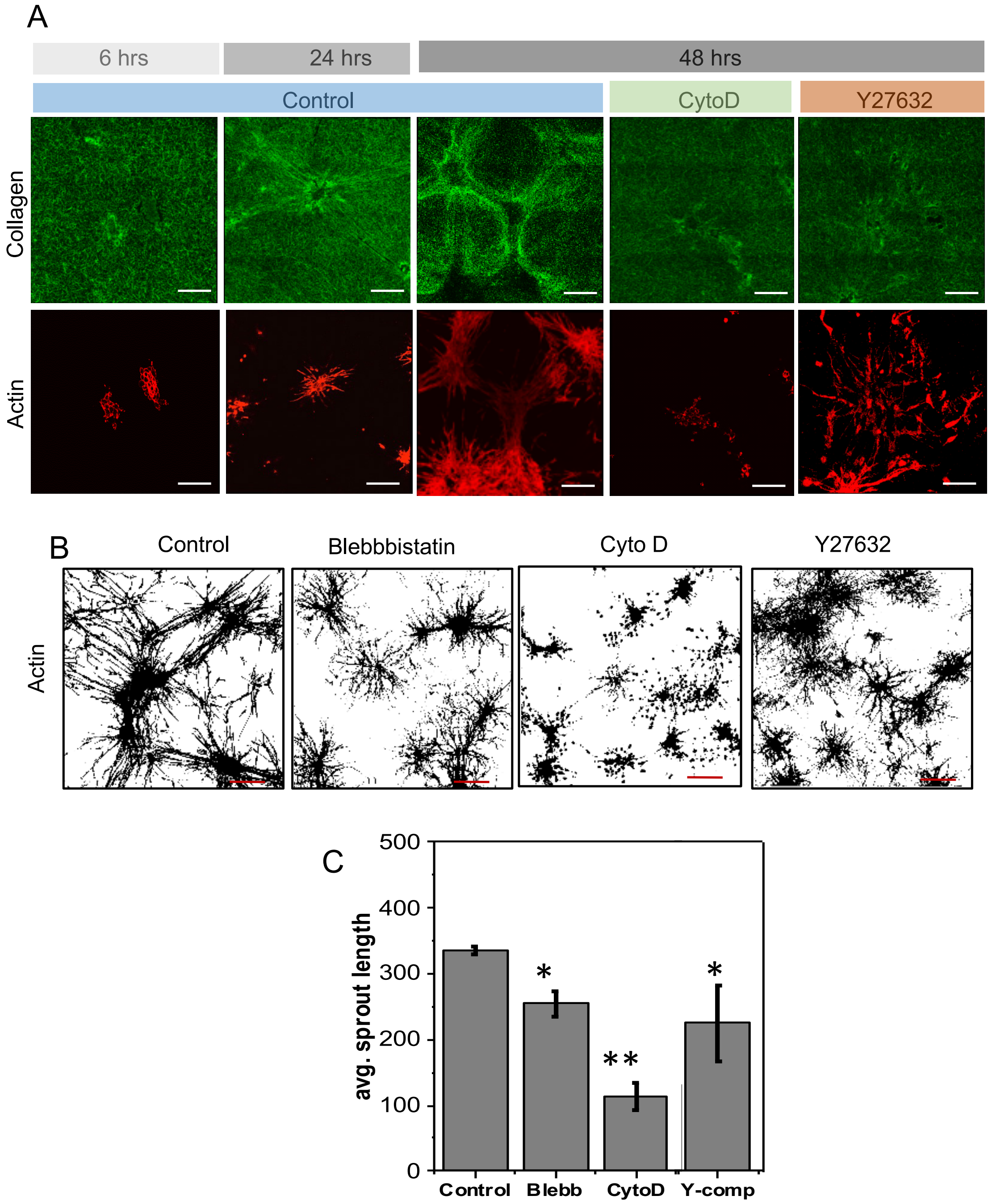

Figure S1. Dependency on cytoskeletal elements to maintain contractility of the fibroblasts in 3D collagen matrix. (A) Representative fluorescence micrographs of immunostained collagen matrix at different time points in sprouting and in response to different cytoskeletal drug treatments. Corresponding phalloidin-stained actin images represent sprouting efficiencies; scale bar: 100μm; (B) Representative inverted images of cells stained with phalloidin, in response to different cytoskeletal drug treatments; scale bar: 100μm; (C) Corresponding quantification of sprouting efficiencies based on average sprout lengths. Error bars represent SD; *P < 0.05; **P < 0.01; Student's t-test.

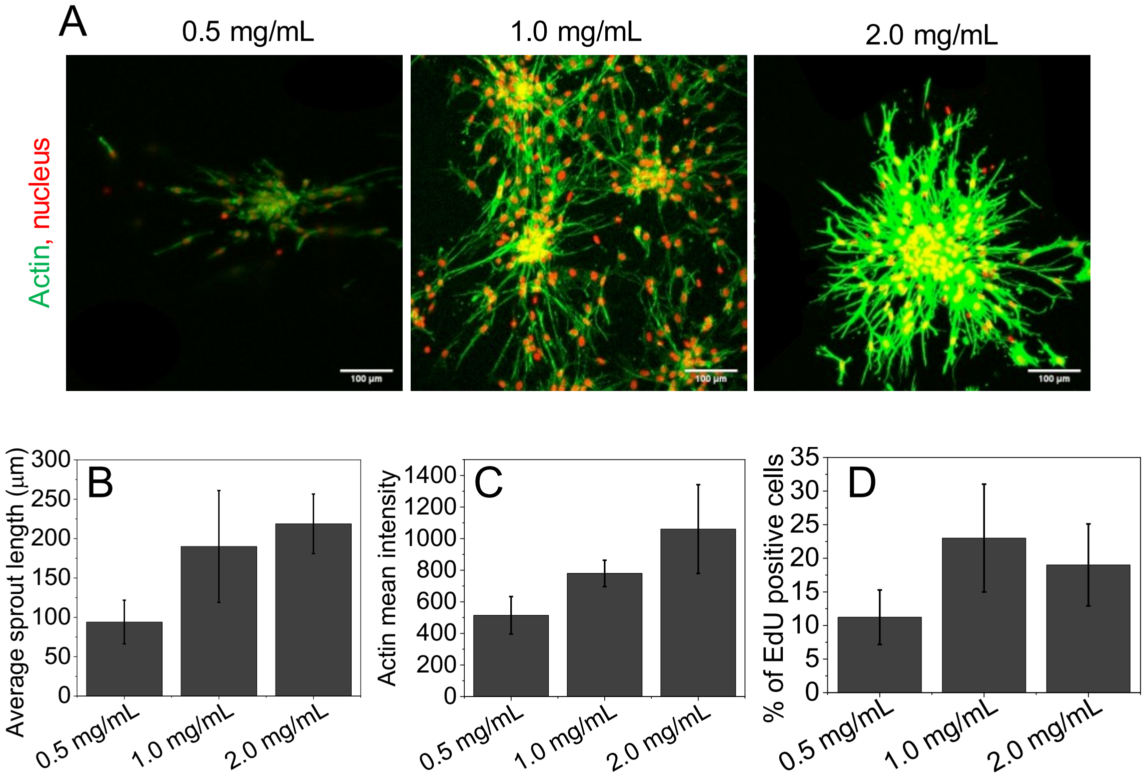

Figure S2. (A) Representative sprouting images of FCG on collagen matrices of varying densities. Cells are stained with phalloidin to label actin; scale bar: 30μm. (B-D) Sprouting efficiencies, contractility, and proliferation levels at varying matrix density measured by average sprout length, mean actin intensities, and percentages of EdU-positive cells in each field of view, respectively. Error bars represent ± SD.

A

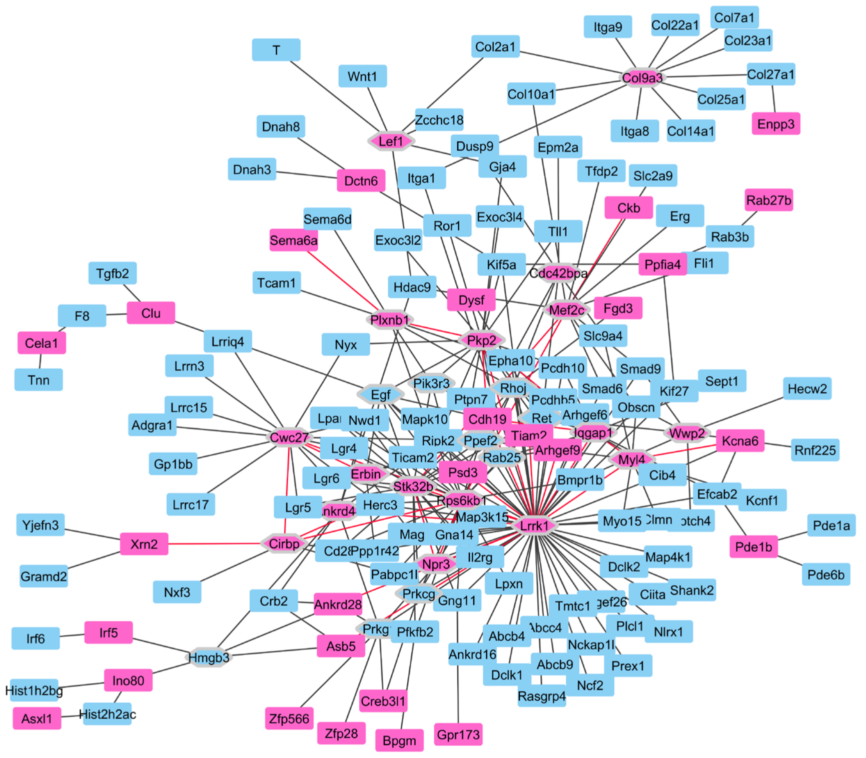

B

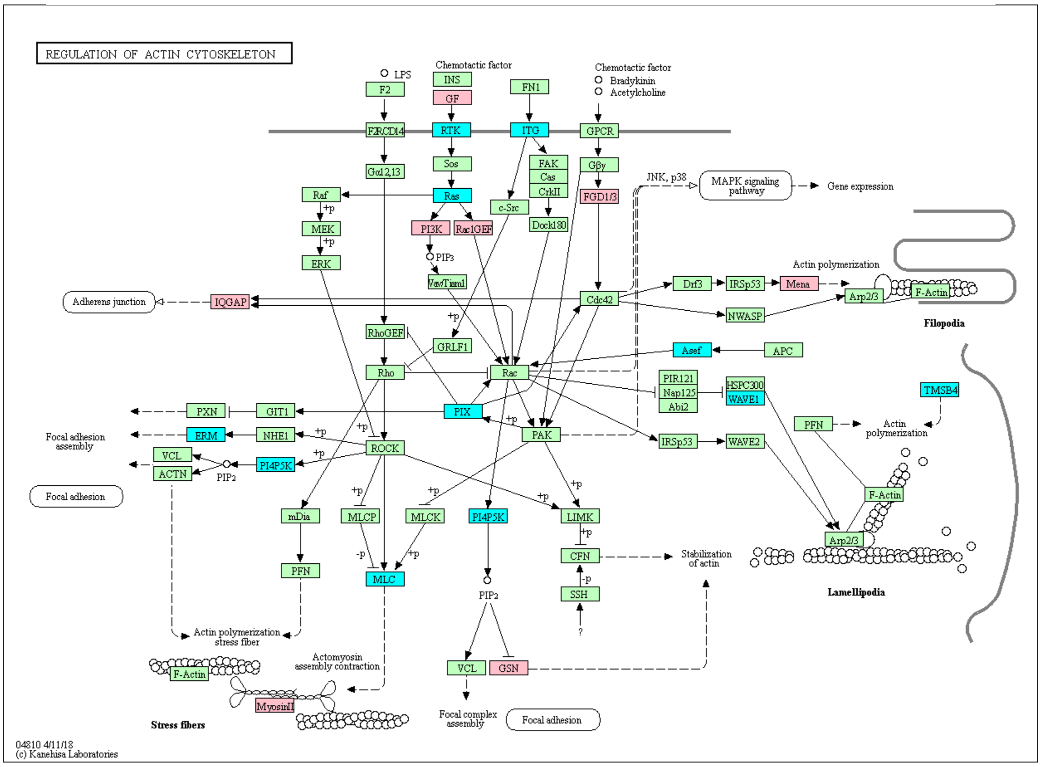

Figure S3. (A) Network analysis providing a functional context to the up-regulated gene list in redifferentiated RF compared to FCG. All genes in the network are up-regulated (Fold change > 1.41) in Fibroblasts', with those in pink representing significant connection nodes (FDR (adjusted p value) < 0.1). Hexagon-shaped genes have many connections to others (with degree higher than 5). (B) KEGG maps for upregulated and downregulated genes in RF and 3T3 clumps embedded in FCG. Pink box refers to the upregulated genes in RF. Cyan box refers to the down regulated genes in RF.

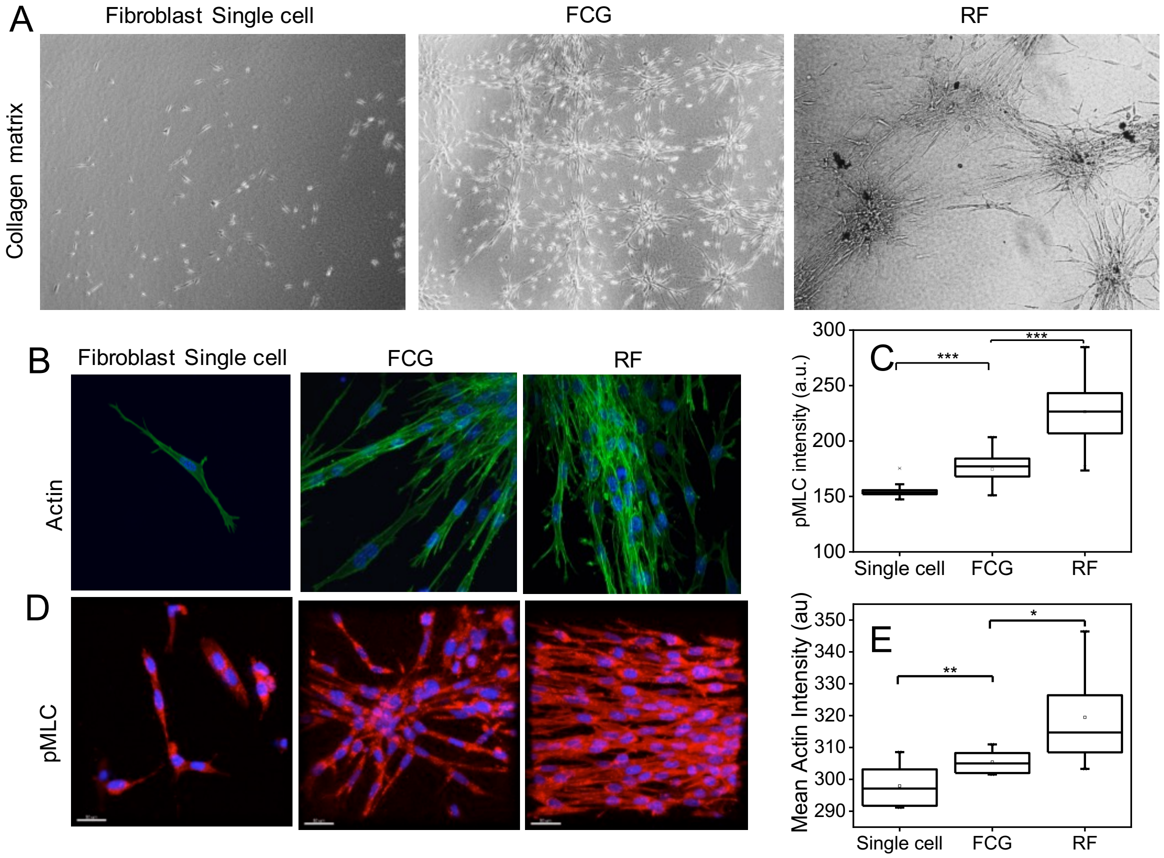

Figure S4. (A) Representative bright field images of cells in the matrix after Matrix incorporated on top of the cells attached on the micropatterns. (B and D) Representative actin and pMLC immunofluorescence micrographs of these three conditions; scale bar: 20μm. (C and E) Corresponding intensity box plots. n=45, 78 and 123 for single cell, FCG and RF conditions, respectively, *P < 0.05; **P < 0.01; ***P < 0.001; Two-sided Student's t-test were used.

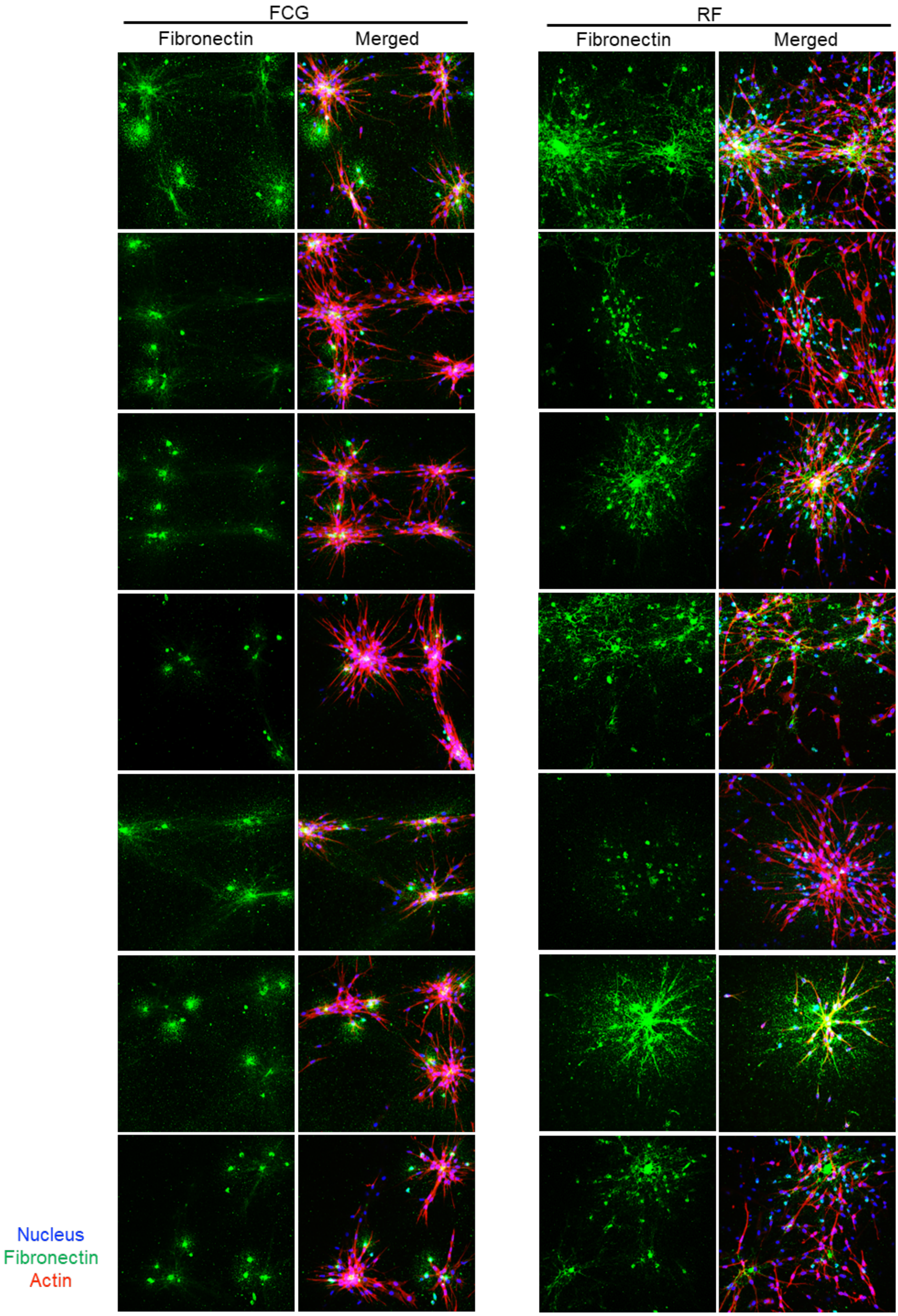

Figure S5. Collage of immunofluorescence micrographs of extracellular fibronectin deposited on to the matrix in these two conditions. In merged images, nucleus is in blue, fibronectin in green and actin in red.

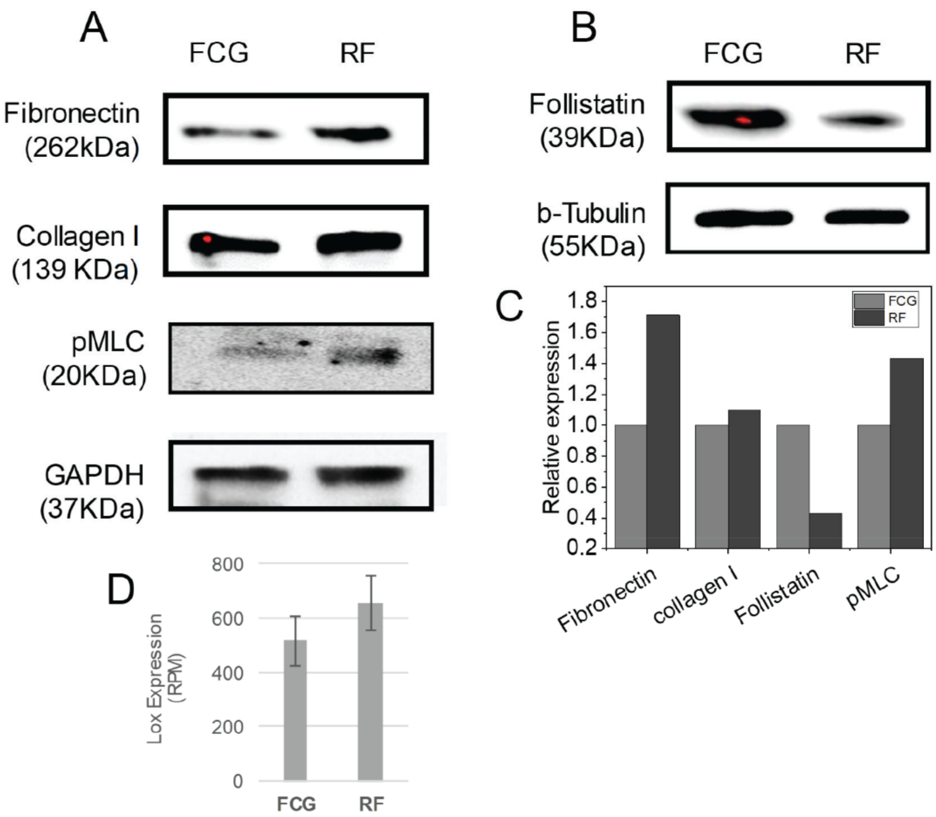

Figure S6. (A and B) Western blots for the selected proteins levels in FCG and RF condition. (C) Quantification plots of the blots for Fibronectin, Collagen I and pMLC with loading control of GAPDH and for Follistatin with loading control of b-Tubulin; (D) Lox expression plot (RNAseq),

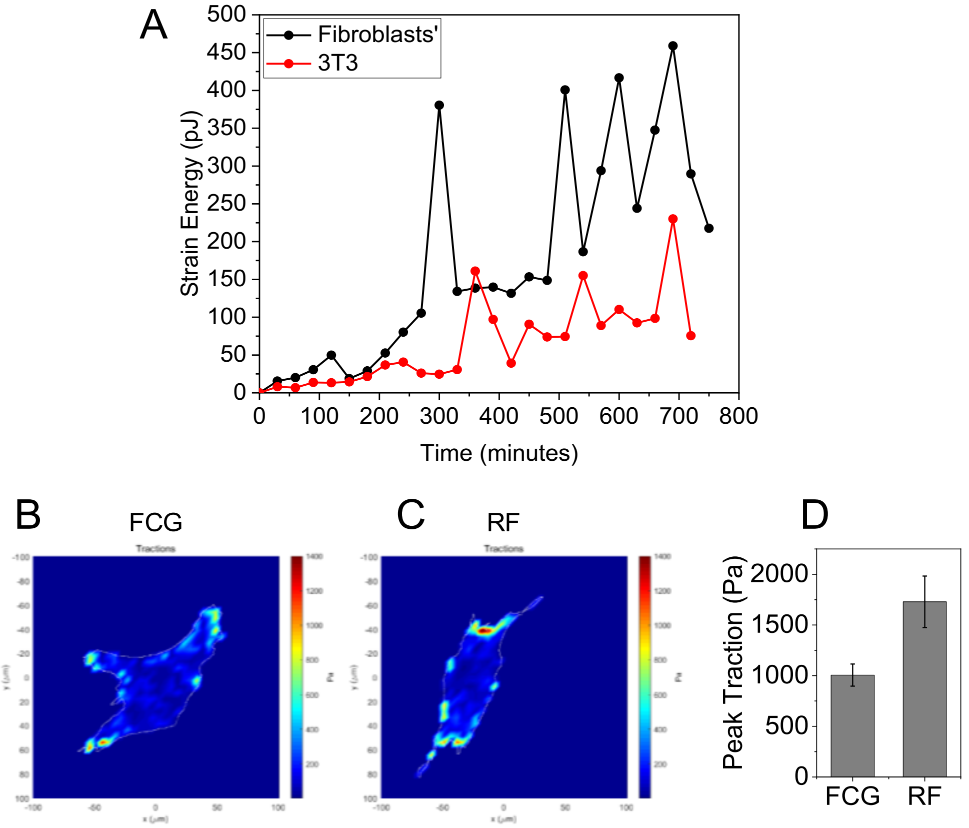

Figure S7. (A) Energy plots depicting strain energy exerted on the matrix by RF and FCG. (B and C) Representative 2D traction map of FCG and RF cells. (C) Peak traction plot of FCG and RF cells.

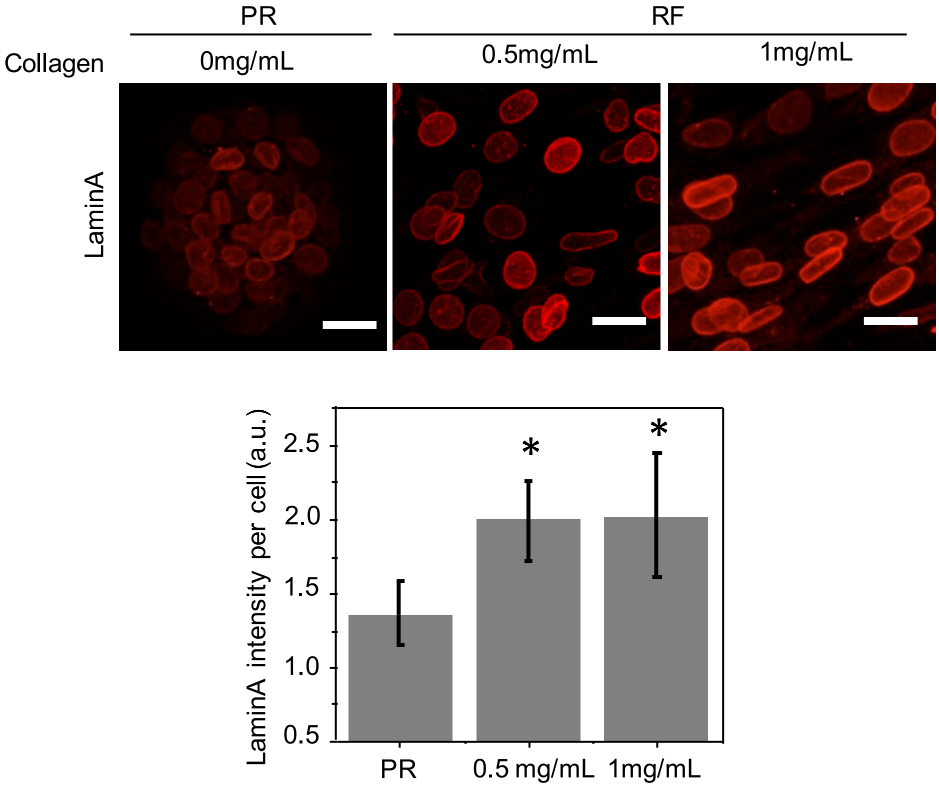

Figure S8. Representative LaminA immunofluorescence micrographs of the nuclei in PR and PR embedded in matrices with varying collagen concentrations (0.5 and 1mg/ml). Scale bar: 20μm. (E) Corresponding bar plots of LaminA intensities. Error bars represent SD; *P < 0.05; Student's t-test.

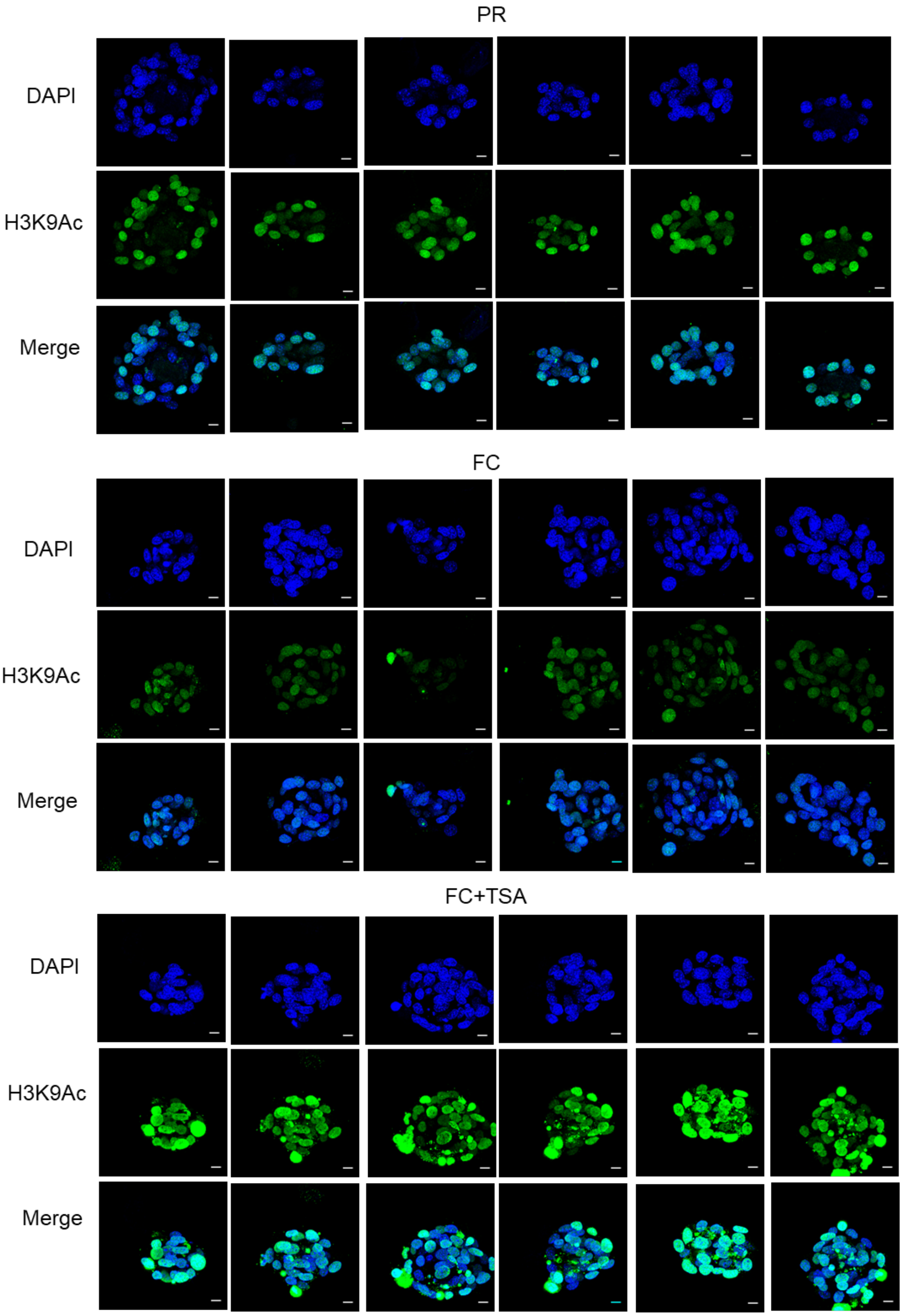

Figure S9. Collage of immunofluorescence micrographs of H3K9Ac levels in the nuclei in partially reprogrammed cell (PR), fibroblast clump (FC) and FC+ TSA treated without gel conditions; scale bar:10μm.

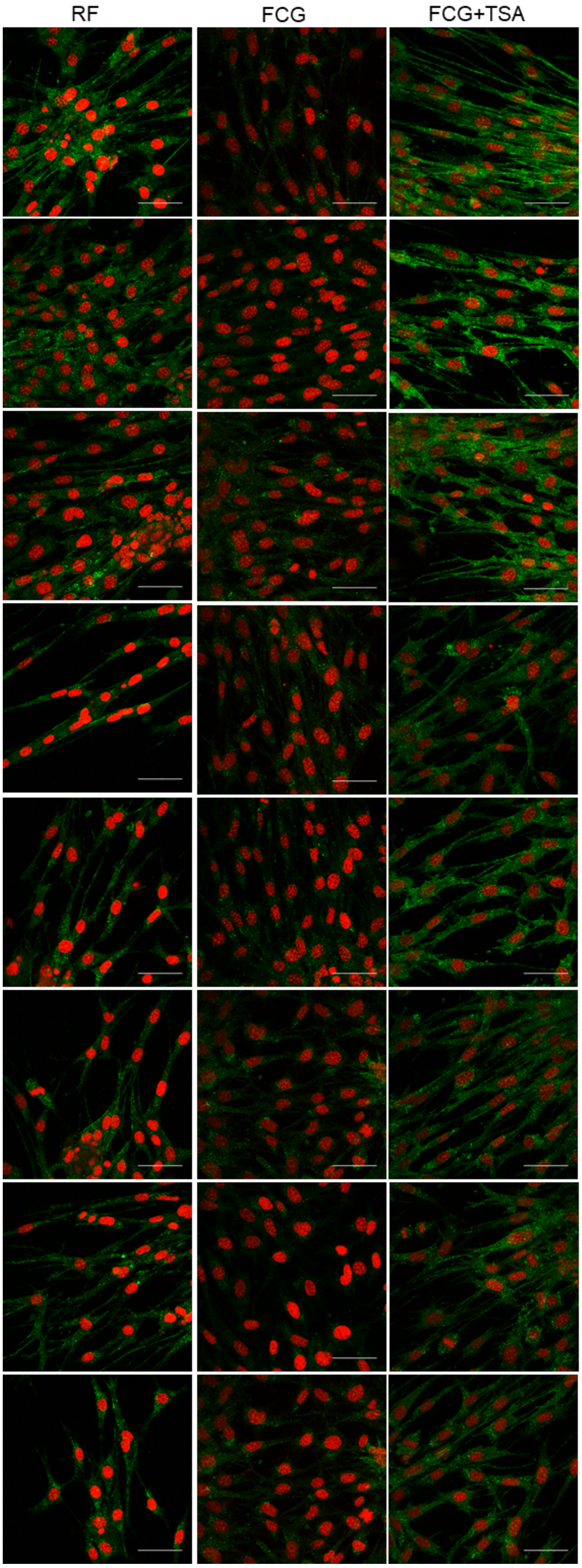

Figure S10. Collage of pMLC immunofluorescence micrographs of the RF, FCG and FCG+TSA-treated cells embedded in collagen matrix. Nucleus is in red; scale bar: 20μm.

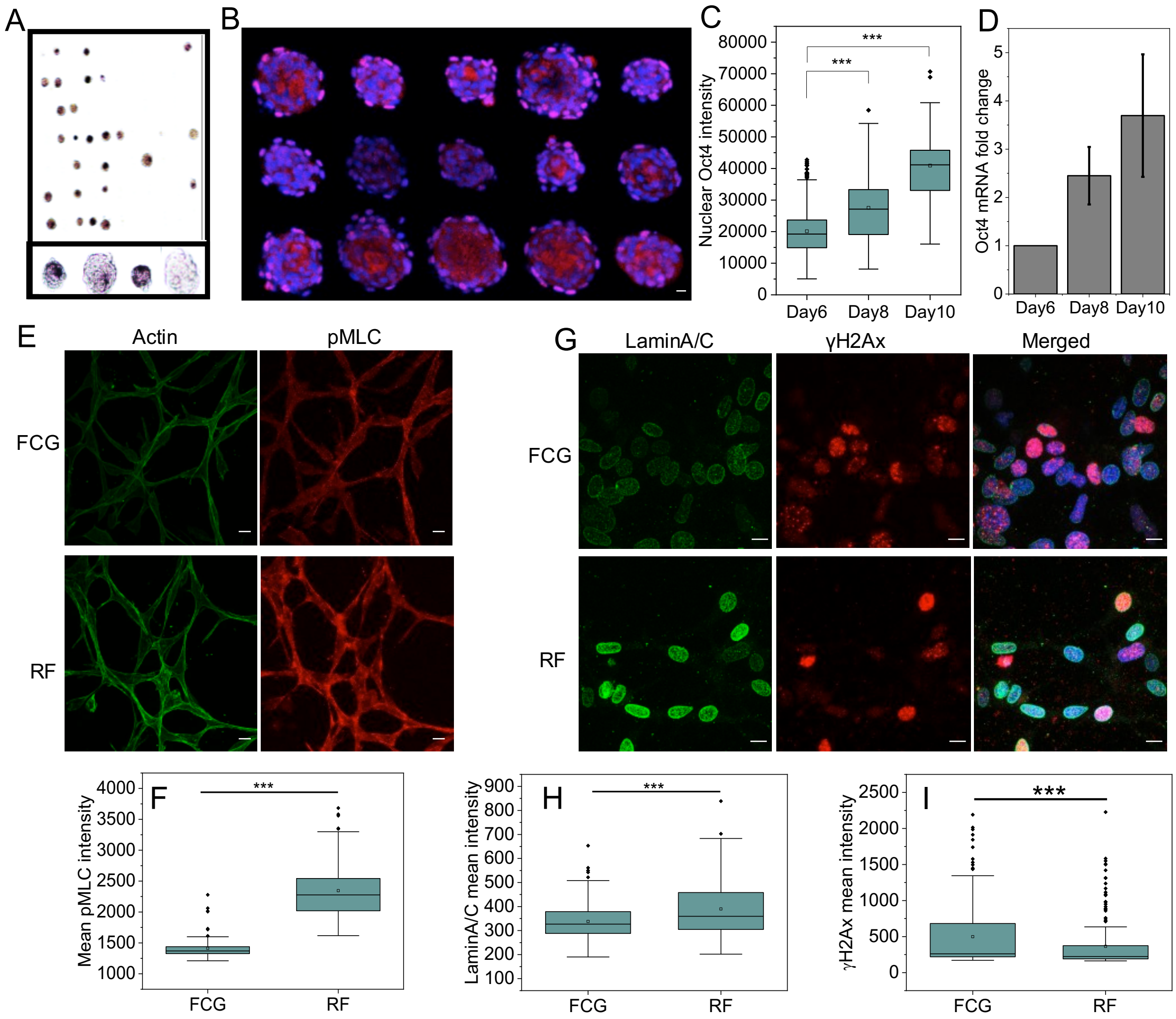

Figure S11. Partial reprogramming and rejuvenation of human foreskin fibroblasts (BJ). (A) Field view of 10days old BJ spheroids with alkaline phosphatase (AP) staining, inset: collage of enlarged spheroids with AP staining; (B) Collage micrograph of BJ spheroids immunostained with Oct4, scale bar: 10μm; (C) Oct4 fluorescence intensity plot of BJ spheroids at day6, day8 and day10. Error bars represent SD; ***P< 0.001; Student's t test. (D) The mRNA level of Oct4 normalized with respect to the mRNA level at day6 BJ spheroids (n = 3 samples). Error bars represent SD. (E) Representative actin and pMLC immunofluorescence micrograph of RF and FCG of BJ cells embedded in 1mg/ml collagen matrix, scale bar:10μm; (F) Corresponding box plots for cellular mean intensity of pMLC for FCG and RF conditions, respectively. ***P < 0.001; two-sided Student's t-test were used. (G) Representative LaminA and gH2AX immunofluorescence micrographs of the nuclei in RF and FCG of BJ cells embedded in 1mg/ml collagen matrix, scale bar:10μm; (H-I) Corresponding box plots of LaminA and gH2AX intensity per nucleus. ***P < 0.001; two-sided Student's t-test were used.

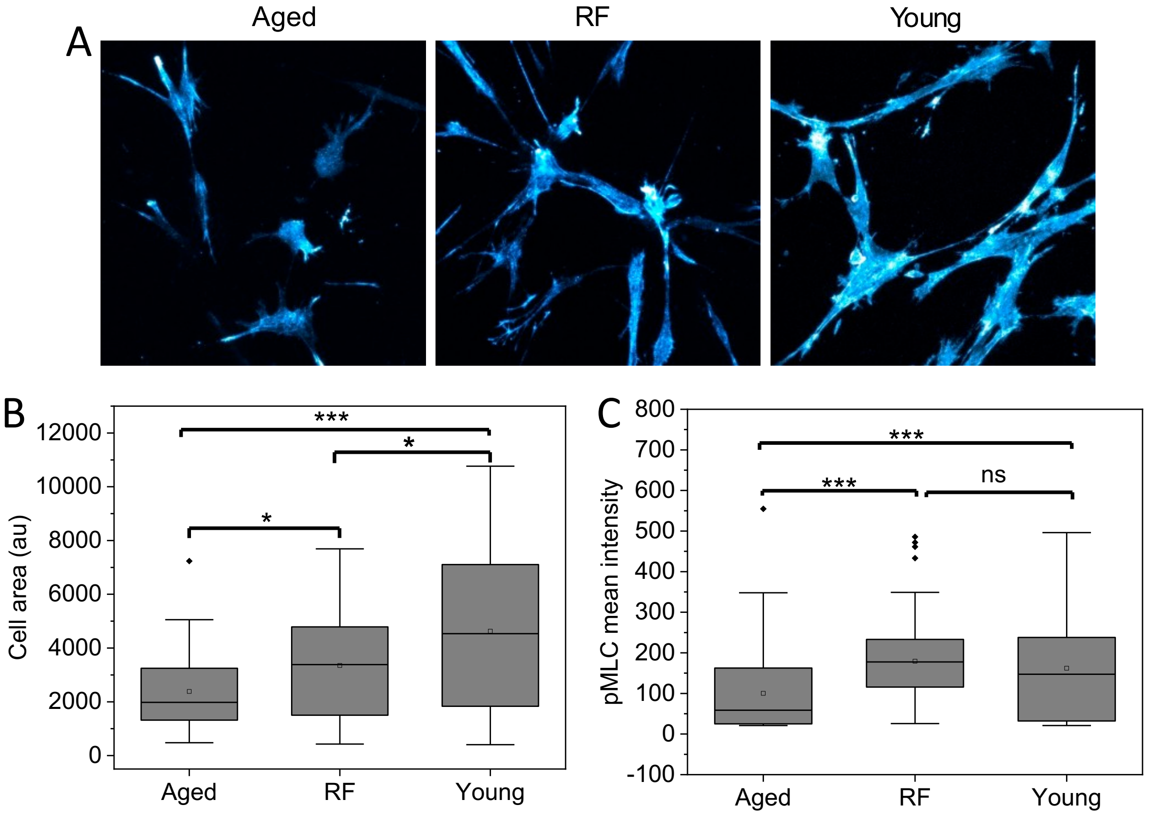

Figure S12. (A) Representative confocal micrographs of aged fibroblasts (GM08401) and rejuvenated (RF) young fibroblasts (GM01652), fibroblast embedded in 3D collagen matrix and immuno-stained with pMLC, (magnification: 400X). (B) Cell area plot of these three type of condition as an assay of cell elongation. Error bars represent ± SD $*P < 0.05$; $***P < 0.001$; two-sided Student's t-test was used. (C) Corresponding box plots for cellular mean intensity of pMLC for aged, RF and young conditions. $***P < 0.001$; two-sided Student's t-test were used.

SUPPLEMENTARY TABLES

Table S1. Primers used for qRT-PCR

gene	forward	backward
Mylpf	TTCAAGGAGGCGTTCACTGTA	TAGCGTCGAGTTCCTCATTCT
Gapdh	GACCAGGTTGTCTCCTGCGACTT	CCATGAGGTCCACCACCCTGTT
Fn1	AAAAGTTTGTGGGAGTCGTTCT	GGCCCTGTTCTTCCATCCAG
Col4a1	CTGGCACAAAAGGGACGAG	ACGTGGCCGAGAATTTCACC
Col1a1	GCTCCTCTTAGGGGCCACT	CCACGTCTCACCATTGGGG
Lamc1	TGCCGGAGTTTGTTAATGCC	CTGGTTGTTGTAGTCGGTCAG
Actb	GGCTGTATTCCCCTCCATCG	GGCTGTATTCCCCTCCATCG
Lmna	GTACAACCTGCGCTCACGCACCGT	CACTGCGGAAGCTTCGAGTGACT

Table S2. Details of primary antibodies

Protein	Supplier name	Catalog number	Species Reactivity	Applications (Immunofluorescence)	Datasheets and Manufacturer's website
gH2AX	Cell Signaling Technologies	2577S	Mouse	1:250 (for overnight at 4C)	https://www.cellsignal.com/products/primary-antibodies/phospho-histone-h2a-x-ser139-antibody/2577
LaminA	Abcam	ab8980	Mouse	1:200 (for overnight at 4C)	https://www.abcam.com/lamin-a-antibody-133a2-ab8980.html
pMLC	Cell Signaling Technologies	3671S	Mouse	1:200 (for 2 days at 4C)	https://www.cellsignal.com/products/primary-antibodies/phospho-myosin-light-chain-2-ser19-antibody/3671
Fibronectin	Abcam	ab2413	Mouse	1:100 (for 2 days at 4C)	https://www.abcam.com/fibronectin-antibody-ab2413.html
Collagen	Abcam	ab6308	Mouse	1:200 (for overnight at 4C)	https://www.abcam.com/collagen-i-antibody-col-1-ab6308.html
H3K9Ac	Abcam	ab12179	Mouse	1:200 (for overnight at 4C)	https://www.abcam.com/histone-h3-acetyl-k9-antibody-ah3-120-chip-grade-ab12179.html

Table S3. The NCBI-SRA accession IDs of public RNAseq data sets used in this study

SRA ID	Description
SRR3083897	E3.5 inner cell mass
SRR3083898	E3.5 inner cell mass
SRR3083901	E4.0 inner cell mass
SRR3083902	E4.0 inner cell mass
SRR3083903	E5.5 epiblast
SRR3083904	E5.5 epiblast
SRR3083905	E5.5 epiblast
SRR3083906	E5.5 epiblast
SRR3083907	E5.5 visceral endoderm
SRR3083908	E5.5 visceral endoderm
SRR3083909	E6.5 epiblast
SRR3083910	E6.5 epiblast
SRR3083911	E6.5 visceral endoderm
SRR3083912	E6.5 visceral endoderm
SRR3083913	E6.5 visceral endoderm
SRR2658589	mESCs from E14
SRR2658612	mESCs from E14
SRR2173784	mESCs from E14 maintained in serum
SRR2173785	mESCs from E14 maintained in serum
SRR2173786	mESCs from E14 maintained in 2i (PD0325901 and CHIR99021)
SRR2173787	mESCs from E14 maintained in 2i (PD0325901 and CHIR99021)
SRR5227280	F123 mESCs
SRR5227281	F123 mESCs
SRR6117986	MEF_Female
SRR6117987	MEF_Female
SRR6117988	MEF_Female
SRR6117992	MEF_Male
SRR6117993	MEF_Male
SRR6117994	MEF_Male
